# Protocadherin 20 maintains intestinal barrier function to protect against Crohn’s disease by targeting ATF6

**DOI:** 10.1186/s13059-023-02991-0

**Published:** 2023-07-05

**Authors:** Shanshan Huang, Zhuo Xie, Jing Han, Huiling Wang, Guang Yang, Manying Li, Gaoshi Zhou, Ying Wang, Lixuan Li, Li Li, Zhirong Zeng, Jun Yu, Minhu Chen, Shenghong Zhang

**Affiliations:** 1 Department of Gastroenterology, The First Affiliated Hospital, Sun Yat-Sen University, 58 Zhongshan II Road, Guangzhou, 510080 Guangdong Province People’s Republic of China; 2 Department of Clinical Laboratory, Henan Provincial People’s Hospital, People’s Hospital of Zhengzhou University, Zhengzhou, 450003 Henan Province People’s Republic of China; 3 People’s Hospital of Henan University, Kaifeng, 475000 Henan Province People’s Republic of China; 4 Department of Minimally Invasive & Interventional Radiology, State Key Laboratory of Oncology in South China, Collaborative Innovation Center for Cancer Medicine, Sun Yat-Sen University Cancer Center, Guangzhou, 510060 Guangdong Province People’s Republic of China; 5 Department of Medical Ultrasonics, Institute of Diagnostic and Interventional Ultrasound, The First Affiliated Hospital of Sun Yat-Sen University, Guangzhou, 510080 Guangdong Province People’s Republic of China; 6 Institute of Digestive Disease and Department of Medicine and Therapeutics, State Key Laboratory of Digestive Disease, Li Ka Shing Institute of Health Sciences, CUHK Shenzhen Research Institute, The Chinese University of Hong Kong, Hong Kong, 999077 People’s Republic of China

**Keywords:** PCDH20, Intestinal barrier integrity, Adherens junctions, Endoplasmic reticulum stress, ATF6, Crohn’s disease

## Abstract

**Background:**

Intestinal barrier dysfunction plays a central role in the pathological onset of Crohn’s disease. We identify the cadherin superfamily member protocadherin 20 (PCDH20) as a crucial factor in Crohn’s disease. Here we describe the function of PCDH20 and its mechanisms in gut homeostasis, barrier integrity, and Crohn’s disease development.

**Results:**

PCDH20 mRNA and protein expression is significantly downregulated in the colonic epithelium of Crohn’s disease patients and mice with induced colitis compared with controls. In mice, intestinal-specific *Pcdh20* knockout causes defects in enterocyte proliferation and differentiation, while causing morphological abnormalities. Specifically, the deletion disrupts barrier integrity by unzipping adherens junctions via β-catenin regulation and p120-catenin phosphorylation, thus aggravating colitis in DSS- and TNBS-induced colitis mouse models. Furthermore, we identify activating transcription factor 6 (ATF6), a key chaperone of endoplasmic reticulum stress, as a functional downstream effector of PCDH20. By administering a selective ATF6 activator, the impairment of intestinal barrier integrity and dysregulation of CHOP/β-catenin/p-p120-catenin pathway was reversed in *Pcdh20*-ablated mice with colitis and *PCDH20*-deficient colonic cell lines.

**Conclusions:**

PCDH20 is an essential factor in maintaining intestinal epithelial homeostasis and barrier integrity. Specifically, PCDH20 helps to protect against colitis by tightening adherens junctions through the ATF6/CHOP/β-catenin/p-p120-catenin axis.

**Supplementary Information:**

The online version contains supplementary material available at 10.1186/s13059-023-02991-0.

## Background

Crohn’s disease (CD) is a chronic recurrent and heterogeneous inflammatory disorder of the gastrointestinal tract that is characterized by segmental ulcers, stenosis, and perforation [1]. Destruction of intestinal barrier integrity is one of the first phases of CD pathogenesis [2], while its repair is the final, yet most important, phase of mucosal healing in CD treatment [3]. Indeed, mucosal healing reduces the long-term adverse outcomes of CD [4]. The apical junctional complex comprises tight junctions, adhesion junctions, and desmosomes, which seal adjacent cells, regulate paracellular transportation, and maintain intestinal barrier function, acting as a dynamic mechanical barrier [5]. In mice, defects in junction-related proteins, such as E-cadherin, p120-catenin, or C1orf106, lead to the development of CD symptoms or enhanced sensitivity to experimental colitis [6–8].

Cadherin genes encode a superfamily of calcium-dependent adhesion-related type-1 transmembrane proteins. Cadherin superfamilies include classical cadherins, protocadherins (PCDHs), and desmosomal cadherins [9]. Moreover, cadherins constitute adherens junctions and participate in the assembly of tight junctions [10]. However, among all cadherins, the expression of PCDH20 is reportedly the most significantly downregulated in patients with CD [11]. PCDH20, also known as PCDH13, is a member of the PCDHδ1 superfamily [12]. Its transmembrane domain is connected to six repeated extracellular cadherin domains and a cytoplasmic peptide tail that lacks conserved motifs. As such, PCDH20 differs from other δ-PCDHs and is predicted to be involved in regulating various signaling pathways [12]. In fact, recent studies have shown that PCDH20 plays key roles in promoting neuron development, synaptogenesis, tumor regulation, and cell signaling [13, 14]. Data from published Genotype-Tissue Expression project indicates that PCDH20 is also expressed in the intestine and colon [15]; however, its role in CD pathogenesis remains unclear.

In this study, we describe a critical role for PCDH20 in maintaining intestinal homeostasis. Intestinal-specific deletion of *Pcdh20* damages epithelial cell proliferation and differentiation and causes morphological changes, including shortened crypts and microvilli. Specifically, the loss of PCDH20 impairs intestinal barrier function by unzipping adherens junctions in mice with colitis via targeting the ATF6/CHOP/β-catenin/p120-catenin axis. Our findings indicate that upregulation of PCDH20 or ATF6 could represent a novel therapeutic strategy for increasing epithelial integrity in CD treatment.

## Results

### PCDH20 is significantly downregulated in the colonic epithelium of CD patients

**Fig. 1:**
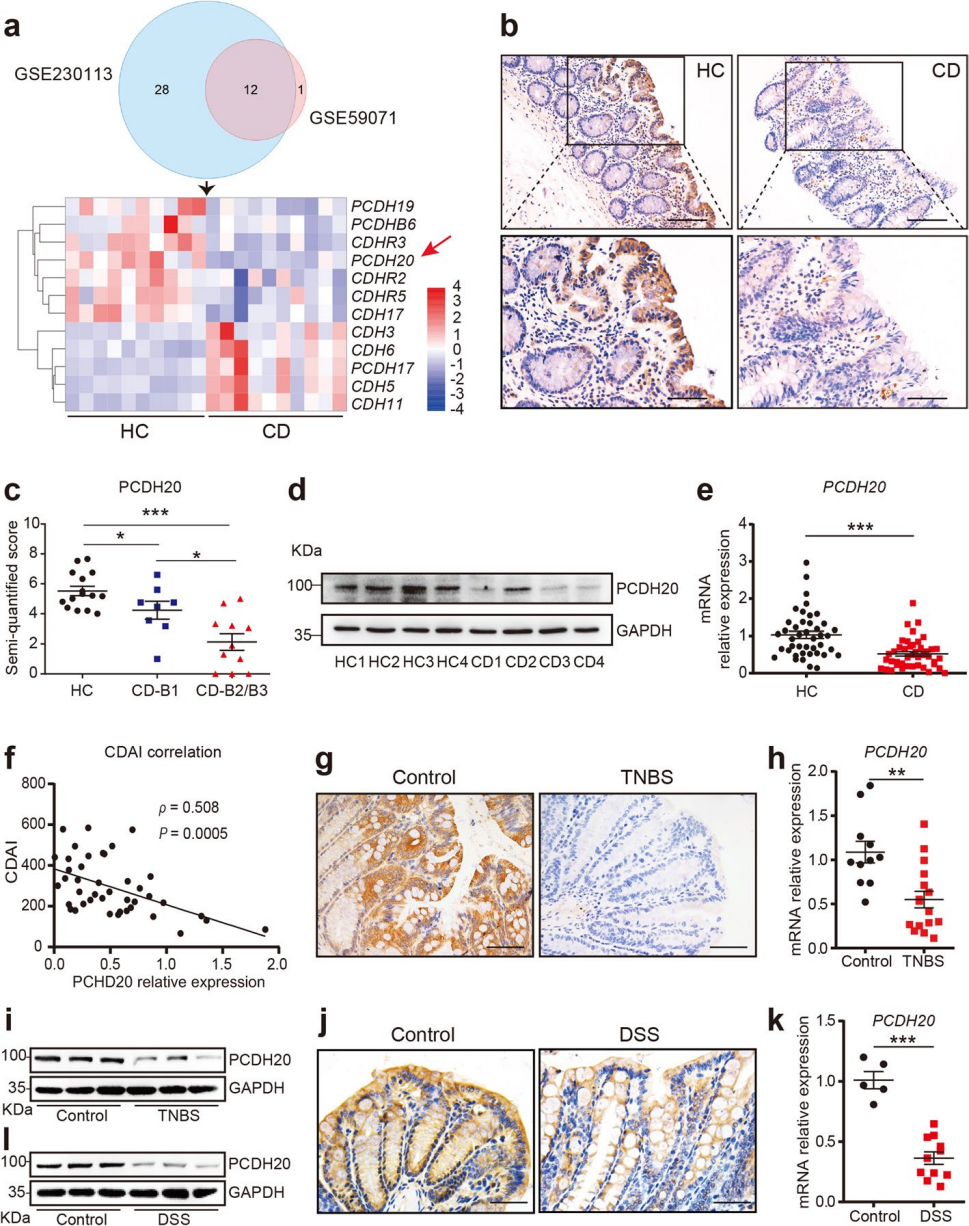
PCDH20 was decreased in the colon epithelium of Crohn’s disease patients and mice colitis models. **a** Venn diagram showing the intersection of cadherins using two different algorithms (top panel). The mRNA expression heatmap of cadherins in colonic epithelium of CD patients (bottom panel). Each group *n* = 8. **b** Representative sections from colonic epithelium of patients with CD (*n* = 19), and HC (*n* = 15) were stained for PCDH20 by using immunohistochemistry. **c** Semi-quantified score of PCDH20 stained by immunohistochemistry in different disease behaviors of CD patients. B1 *n* = 8, B2/3 *n* = 11. **d** Immunoblots of PCDH20 protein in the colonic mucosa of CD patients. CD *n* = 8, HC *n* = 8, representative image. **e** RT-qPCR detection of *PCDH20* mRNA expression in colon biopsy samples. CD *n* = 43, and HC *n* = 42. **f** Correlation between *PCDH20* mRNA expression and CDAI in patients with CD (*n* = 43). **g–i** Immunohistochemistry staining (**g**), RT-qPCR (**h**), and immunoblots (**i**) for PCDH20 levels in colonic epithelium of TNBS-induced colitis mice model. Each group *n* = 3 (**g, i**). TNBS *n* = 16, control *n* = 11 (**h**). Representative images for immunohistochemistry staining. **j–l** Immunohistochemistry staining (**j**), RT-qPCR (**k**), and immunoblots (**l**) for PCDH20 levels in colonic epithelium of DSS-induced colitis mice models. Each group *n* = 3 (**j, l)**, DSS *n* = 11, control *n* = 5 (**k**); representative images for immunohistochemistry staining. The data in **c**, **e**, **h**,** k** were presented as mean ± SEM. * *p* < 0.05, **p < 0.01 vs. Control, ***p < 0.001 vs Control. Magnification × 200 (top in **b**) and × 400 (bottom in **b**, **g**, **j**). Scale bars = 100 μm (top in **b**) and 5 μm (bottom in **b**, **g**, **j**). # Gene expression in qPCR was normalized to beta-actin in each group. Abbreviations: Crohn’s disease, CD; healthy control, HC; non-stricture, non-penetrating behavior, B1; stricture behavior, B2; penetrating behavior, B3; Crohn’s disease activity index, CDAI

To identify key cadherins involved in CD pathogenesis, colonic mucosa from 10 pairs of CD patients and healthy donors were obtained and performed mRNA sequencing to get dataset GSE230113 [16], with clinical characteristics of selected subjects in Additional file 1: Table S1. The integration of GSE230113 with the published mRNA microarray sequence dataset (GSE59071 [11], Additional file 1: Fig. S1) identified 12 candidate cadherins (Fig. 1a). We found that PCDH20 was significantly downregulated in the colonic epithelium of almost all patients with CD (Fig. 1a). Immunohistochemistry staining revealed that PCDH20 protein was primarily located in the epithelium, particularly in the brush border of healthy human colon samples, while its levels were decreased in the inflamed colon epithelium of patients with CD compared to those in the colon epithelium of healthy controls (Fig. 1b). Moreover, PCDH20 protein expression in inflamed mucosa was markedly downregulated in patients with CD exhibiting stenosis and penetrating phenotypes, as compared to those exhibiting an inflammatory phenotype (Fig. 1c). Expression of PCDH20 mRNA and protein was also significantly decreased in the inflamed epithelium of CD patients compared with the epithelium of healthy controls, as determined via Western blotting and real-time qPCR (Fig. 1d,e). Additionally, *PCDH20* mRNA expression was significantly low in inflammatory sites compared to non-inflammatory sites of the colonic mucosa in CD patients (Additional file 1: Fig. S2a). We also evaluated the relationship between PCDH20 and CD disease severity and found that *PCDH20* mRNA expression was negatively correlated with CDAI in the inflamed mucosa of CD patients (Fig. 1f).

### PCDH20 is significantly downregulated in the colonic epithelium of mice with colitis

To further verify our findings, we constructed DSS- and TNBS-induced colitis models by analyzing weight loss (Additional file 1: Fig. S2b, d), colon length (Additional file 1: Fig. S2c, f), DAI (Additional file 1: Fig. S2e), and MPO (Additional file 1: Fig. S2g). Using immunohistochemistry (Fig. 1g, j), real-time qPCR (Fig. 1h, k), and immunoblot (Fig. 1i, l), we detected a decreased expression of PCDH20 in both colitis groups compared to that in the control groups.

### PCDH20 maintains epithelial morphology and microbial balance by regulating cell proliferation and differentiation

**Fig. 2:**
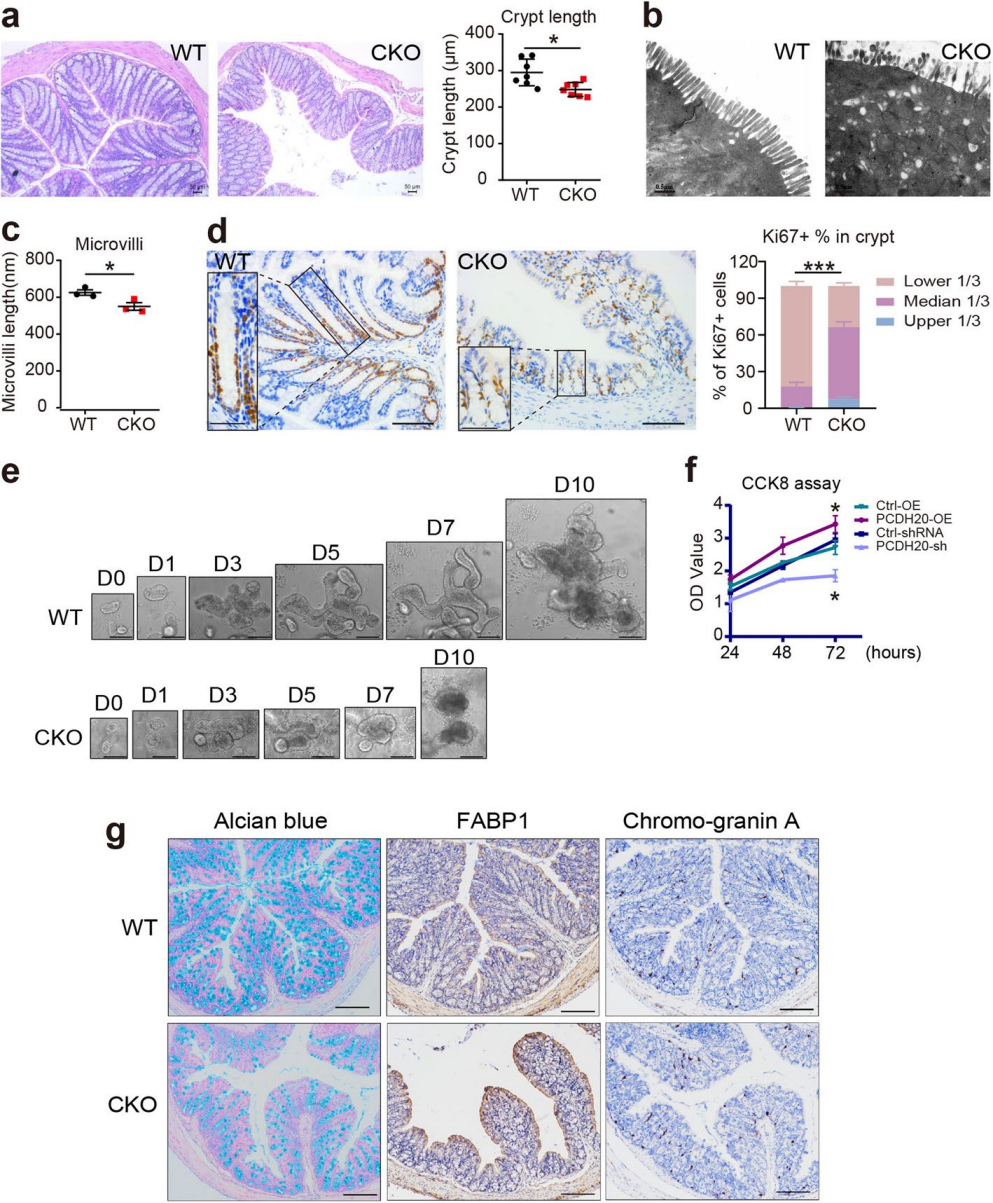
PCDH20 maintained epithelial proliferation, differentiation, and morphology. **a** *Pcdh20* CKO mice presented shortened crypts in colon stained with H&E. Each group *n* = 7. Magnification × 100, scale bars = 50 μm. **b,c** Shorten microvilli observed with transmission electron microscope in *Pcdh20* CKO mice compared with WT. *n* = 3 for each group. Magnification × 18,500, scale bars = 0.5 μm. **d** Representative images of ki67 stained with immunohistochemistry in colon mucosa of *Pcdh20* CKO and WT mice. Each group *n* = 3. Magnification × 200 (right) and × 400 (left), scale bars = 100 μm (right) and 50 μm (left). **e** Representative images of intestinal organoid growth in *Pcdh20* CKO and WT mice. Each group *n* = 3. Magnification × 100, scale bars = 500 μm. **f** Cell count kit-8 (CCK8) assay in Caco-2 cell line with stably *PCDH20* overexpression and knockdown. Three independent biological replicates. **g** Representative images of differentiated cells in the colon epithelium of *Pcdh20* CKO mice (Alcian blue, goblet cells; FABP1, enterocytes; Chromo-graninA, enteroendocrine cells). Each group *n* = 3. Magnification × 100, scale bars = 150 μm. The data in **a**, **c**, **f** were presented as mean ± SEM. * *p* < 0.05, vs. WT. Intestinal epithelial conditional knockout, CKO; wild-type, WT

We generated CKO mice to study the role of PCDH20 in the gut. The deletion of PCDH20 in the colon epithelium of *Pcdh20* CKO mice was confirmed by RT-PCR (Additional file 1: Fig. S3a) and immunoblot (Additional file 1: Fig. S3b). Significant changes were observed in 8-week-old *Pcdh20* CKO mice with respect to epithelial morphology, but not in weight or stool consistency (data not shown). The crypt depth in the colon (Fig. 2a) and the length of the villi in the small intestine (Additional file 1: Fig. S3c) were both significantly decreased in *Pcdh20* CKO mice compared with those of WT mice. We also observed significantly shorter microvilli in the colonic epithelium of *Pcdh20* CKO mice than that of WT mice (Fig. 2b, c), using transmission electron microscopy (TEM) analysis, while tight junctions (Additional file 1: Fig. S3d) remained intact in the *Pcdh20* CKO mice. These data suggest that PCDH20 is required for the maintenance of epithelial morphology.

We then performed immunohistochemical staining to assess proliferation to identify the cause for the observed morphological changes in *Pcdh20* CKO mice. The results showed that significantly fewer Ki67 + proliferative cells were observed in the colonic epithelium of *Pcdh20* CKO mice than WT mice (Fig. 2d). Moreover, Ki67 + proliferating cells in *Pcdh20* CKO mice were primarily localized within the median third of the crypt, while those of WT mice were within the low one third (Fig. 2d). We then constructed the intestinal organoids of *Pcdh20* CKO and WT mice in vitro to visualize the effect of PCDH20 on epithelial proliferation. Beginning on day 5 of the culture, we observed fewer buddings and smaller *Pcdh20* CKO intestinal organoids (Fig. 2e), indicating impaired epithelial proliferation. To further characterize the effect of PCDH20 in epithelial proliferation, stable *PCDH20-*overexpressing and knockdown colonic Caco-2 cells were established (Additional file 1: Fig. S4a-d). The CCK8 assay revealed significantly fewer proliferative cells in the *PCDH20*-knockdown Caco-2 cells than that in the control cells, while more proliferative cells were observed in the *PCDH20*-overexpressing Caco-2 cells than that in control cells (Fig. 2f). These data suggest that PCDH20 is required for the maintenance of epithelial proliferation.

To determine whether *PCDH20* knockdown affects epithelial differentiation, we conducted immunohistochemical staining for specific cellular markers in the epithelial cells of the colon and ileum. In these regions of the *Pcdh20* CKO mice, fewer goblet cells, as well as more enterocytes and enteroendocrine cells, were observed (Fig. 2g and Additional file 1: Fig. S5-6). Moreover, significantly fewer Tuft cells were observed in the ileum of *Pcdh20* CKO mice than that of WT mice (Additional file 1: Fig. S6). These data suggest that PCDH20 is required for intestinal differentiation.

Decreased goblet cells in *Pcdh20* CKO mice might interfere with the balance of gut flora, primarily due to the reduced secretion of mucin. To gain more insight into the function of PCDH20 on the microbiota, fecal metagenomic sequencing was conducted in *Pcdh20* CKO mice. At the species level, knocking out PCDH20 decreased the relative abundance of Bacteroides sp.CAG:927 and Prevotella sp.CAG:485, while increasing the relative abundance of Akkermansia muciniphila, Pseudomonas, Fidobacteria, and Lachnospira A4 (Additional file 1: Fig. S7a-dd). Alpha and beta diversity analysis found that PCDH20 deletion deregulated the gut microbiota (Additional file 1: Fig. S7e-f). Further analysis shows that Lachnospira, Ruminococcus, Unclassified Clostridium, Clostridium, and Unclassified Firmicutes contributed the most to the dysbiosis after PCDH20 deletion (Additional file 1: Fig. S8). KEGG enrichment pathway analysis showed that intestinal microbial differentially expressed genes in *Pcdh20* CKO mice were mainly enriched in several signaling pathways, such as biosynthesis of other secondary metabolites, amino acid metabolism, lipid metabolism, gene transcription, and membrane transport (Additional file 1: Fig. S9). These data suggest that PCDH20 is required for the balance of gut microbiota.

Collectively, these data indicate that PCDH20 maintains epithelial morphology and microbial balance by regulating the proliferation and differentiation of intestinal epithelial cells.

### Loss of epithelial PCDH20 aggravates intestinal barrier function and severity in colitis in vivo and in vitro

**Fig. 3:**
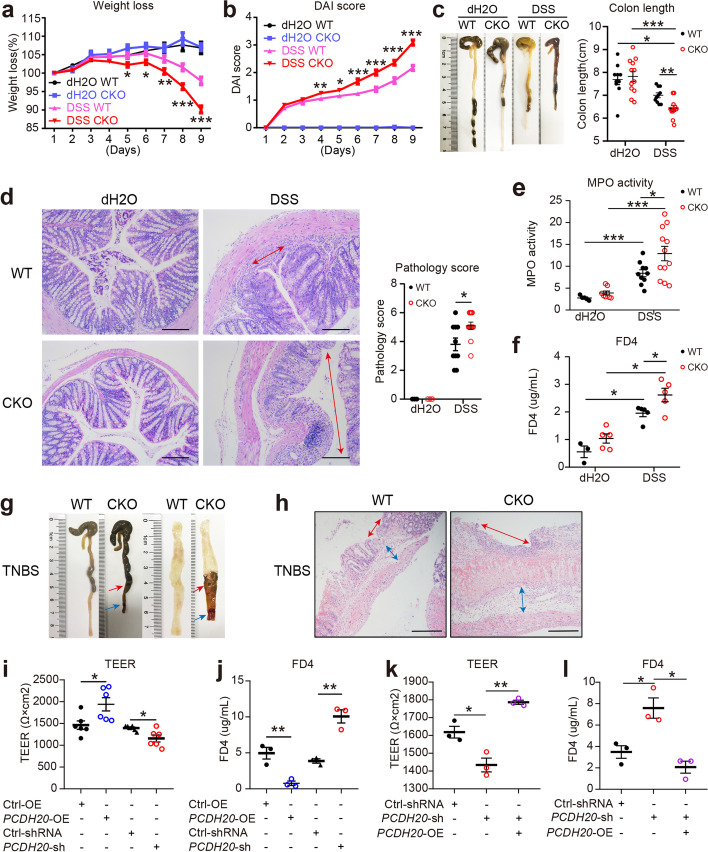
PCDH20 deficiency impaired intestinal barrier integrity in colitis. **a–e** *Pcdh20* CKO mice developed more severe colitis than WT mice after DSS administration. Weight loss (**a**), disease activity index (**b**), colon length (**c**), ulcer and pathological score (**d**), and myeloperoxidase (MPO) activity (**e**). **f** Increased FD4 level detected in the serum of *Pcdh20* CKO mice with DSS-induced colitis. **g,h** *Pcdh20* CKO mice developed more severe colitis than WT mice after TNBS administration. Shortened colon length, perforation (red arrow), stenosis (blue arrow) (**g**), greater ulcers, and edema (**h**). **i,j** Greater paracellular permeability in *PCDH20*-deficient Caco-2 cells treated with TNFα (2.5 ng/mL) and IFN-γ (10 ng/mL) compared with control. Transepithelial electrical resistance (TEER) (**i**), paracellular permeability assessment with FD4(**j**). **k,l** Paracellular permeability in *PCDH20*-dificient Caco-2 cells rescued by transfecting PCDH20 plasmid. TEER (**k**), paracellular permeability assessment with FD4 (**l**). Magnification (**d–h**) × 100, scale bars = 100 μm. The data in **a**-**f**, **i**-**l** were presented as mean ± SEM. * *p* < 0.05, **p < 0.01, ***p < 0.001 vs Control. Numbers in each group: **a,b** dH2O-WT *n* = 10, dH2O-CKO *n* = 12, DSS-WT *n* = 22, DSS-CKO *n* = 26; **c** dH2O-CKO *n* = 12, DSS-CKO *n* = 11, other groups *n* = 10; **d** dH2O-WT *n* = 3, dH2O-CKO *n* = 3, DSS-WT *n* = 10, DSS-CKO *n* = 12; **e** dH2O-WT *n* = 5, dH2O-CKO *n* = 8, DSS-WT *n* = 10, DSS-CKO *n* = 12; **f** dH2O-WT *n* = 3, other groups *n* = 5; **g,h** Each group *n* = 3. Intestinal epithelial conditional knockout, CKO; wild-type, WT; overexpression, OE

Impaired epithelium morphology can significantly contribute to CD pathogenesis [7, 17]. Therefore, to further investigate whether PCDH20 plays such a role, *Pcdh20* CKO mice were subjected to DSS-induced colitis. At day 5 of DSS treatment, *Pcdh20* CKO mice began to exhibit significant weight loss (Fig. 3a), a higher DAI (Fig. 3b), and shortened colon length (Fig. 3c) compared to the WT controls. Moreover, DSS-treated *Pcdh20* CKO mice had more severe ulcers, significantly higher pathological scores (Fig. 3d), and greater infiltration of neutrophils in the colon (Fig. 3e). Additionally, by quantifying the amount of FD4 to pass through the epithelial barrier, we detected significantly increased intestinal permeability in *Pcdh20* CKO mice compared with WT mice (Fig. 3f). These findings indicate that PCDH20 deficiency impairs the intestinal barrier and results in more severe inflammation in DSS-induced colitis.

To further clarify these findings, we used TNBS to induce colitis in *Pcdh20* CKO mice and observed similar results. In the TNBS-treated group, we detected significant weight loss (Additional file 1: Fig. S10a), colonic edema, stenosis, and perforation and shortened colons (Fig. 3g and Additional file 1: Fig. S10b) in *Pcdh20* CKO mice compared with WT mice. Moreover, the colons of *Pcdh20* CKO mice exhibited more severe mucosal barrier damage, including giant ulcers (Fig. 3h, red arrow), as well as greater infiltration of inflammatory cells, more severe coagulative necrosis in the epithelium (Fig. 3h), and more acute edema in the lamina propria (Fig. 3h, blue arrow). In the TNBS group, MPO analysis revealed that more neutrophils infiltrated the colon of *Pcdh20* CKO mice than WT mice (Additional file 1: Fig. S10c). Further, we detected significantly increased intestinal permeability in *Pcdh20* CKO mice, compared with WT mice (Additional file 1: Fig. S10d). These data indicate that PCDH20 deficiency aggravates the intestinal barrier and causes ulcer formation in mice with TNBS-induced colitis.

To further elucidate the effect of PCDH20 on the epithelial barrier, we measured TEER and FD4 to assess barrier function in monolayers of Caco-2 cells with stable overexpression and knockdown of *PCDH20*. Maximal TEER (Fig. 3i) and minimal FD4 (Fig. 3j) were detected in *PCDH20*-overexpressing cells compared with control cells, while a reduction in TEER (Fig. 3i) and increase in FD4 (Fig. 3j) was observed in *PCDH20*-deficient cells compared with the scramble control. Moreover, transfection of *PCDH20*-deficient cells with a *PCDH20* plasmid reversed the reduced TEER (Fig. 3k) and increased FD4 (Fig. 3l). Though proliferation defect existed in *PCDH20*-deficient cell lines, it did not interfere with TEER differences (Additional file 1: Fig. S11). These findings indicate that PCDH20 plays a crucial role in maintaining in vitro epithelial barrier function. Collectively, these data demonstrate that PCDH20 deficiency impairs epithelial barrier integrity in vivo and in vitro.

### PCDH20 deficiency induces impaired epithelial barrier integrity by unzipping adherens junctions

**Fig. 4:**
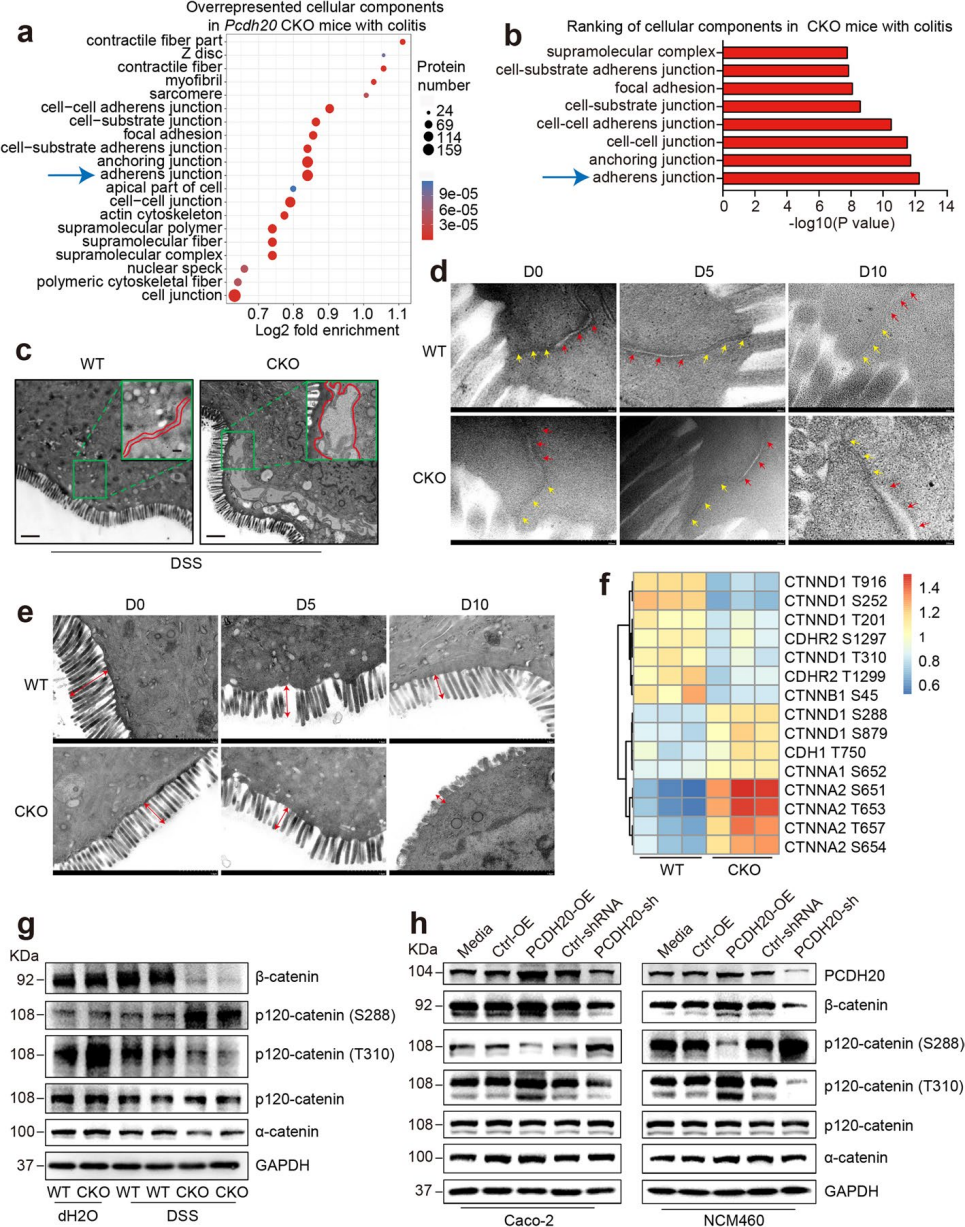
PCDH20 deficiency impaired epithelial barrier integrity in colitis by disrupted adherens junctions. **a,b** Gene ontology (GO) terms of the top regulated cellular components in phosphoproteomics of colon of *Pcdh20* CKO mice with DSS-induced colitis (*n* = 3). Bubble graph of top 20 cellular components (**a**), adherens junctions ranked first in top 8 cellular components (**b**). **c–e** Distinct gap in adherens junction (**c**, red arrows in **d**), but not tight junction (yellow arrows in **d**) and shorten microvilli (**e**, red arrows) in the colon epithelium of *Pcdh20* CKO mice at day 10 after DSS administration, observed by transmission electron microscope. Magnification: × 3000 (**c**), × 10,000 (zooming box, **c**), × 40,000 (**d**), × 8000 (**e**); scale bars = 1 μm (**c**, **e**) and 200 nm (zooming box in **c, d**). Representative images, *n* = 3. **f** Phosphoproteomics analysis on the phosphorylation sites of adherens junctions proteins in *Pcdh20* CKO mice with DSS-induced colitis (*n* = 3). **g,h** Immunoblots of adherens junctions components in *Pcdh20* CKO mice with DSS-induced colitis (**g**) and cell lines with stable *PCDH20* knockdown and overexpression under the treatment of TNFα (2.5 ng/mL) and IFN-γ (10 ng/mL) (**h**)

Phosphorylation plays a crucial role in intestinal inflammation and barrier function in inflammatory bowel disease [18–20]. Thus, to identify the potential mediators of PCDH20 on epithelial barrier integrity, LC–MS/MS technology featuring proteomics and phosphoproteomics were applied using the inflamed colon of *Pcdh20* CKO and WT mice. Quality control analysis (Additional file 1: Fig. S12a-c) indicated that the reproducibility between biological replicates in the proteomics and phosphoproteomics analysis systems was robust. Among 15,349 phosphosites in 4184 proteins identified, 1180 were downregulated and 455 were upregulated with more than a 1.5-fold change (Additional file 1: Fig. S12d). This indicates that phosphorylated proteins played an important role in PCDH20-mediated functions. Further functional enrichment analysis on phosphoproteomics revealed that the most enriched “cellular component” aspect was adherens junctions (Fig. 4a, b), while regulation of cytoskeleton organization and actin filament organization ranked top in the “biological process” aspect (Additional file 1: Fig. S12e). Moreover, complicated network of phospho-proteins involved in adherens junctions was found (Additional file 1: Fig. S12f). Considering that cytoskeleton organization and actin filament organization both contribute to the function of adherens junctions [21], we hypothesized that PCDH20 deficiency impairs intestinal barrier integrity by regulating adherens junctions.

To test our hypothesis, we conducted a TEM observation of the epithelial ultrastructure of *Pcdh20* CKO and WT mice treated with DSS. A significant gap along the entire basolateral surface was observed in *Pcdh20* CKO epithelial cells on day 10 (Fig. 4c, d), but not day 5 (Fig. 4d and Additional file 1: Fig. S13a) following DSS treatment. However, the tight junctions remained intact (Fig. 4d, yellow arrow), indicating that *Pcdh20* CKO selectively targeted the adherens junctions during inflammation. Moreover, shortened microvilli were observed in the epithelial cells of *Pcdh20* CKO mice on day 10 post-DSS treatment (Fig. 4e and Additional file 1: Fig. S13b). These results indicate that PCDH20 deficiency impairs intestinal barrier function by disconnecting adherens junctions in the late stage of colitis, which is consistent with our functional enrichment analysis on phosphoproteomics and supports our hypothesis.

Adherens junctions consist of cadherins and catenin complexes, including α-catenin, β-catenin, and p120-catenin. Thus, we analyzed the proteins associated with these junctions using the phosphoproteomics data that showed more than a 1.2-fold change and a *p* value < 0.05. We found that 80% of the differential phosphorylation occurred in the catenin complex while half of it occurred in p120-catenin (Fig. 4f). Immunoblotting further revealed that in the colitis models, β-catenin was significantly downregulated, while levels of α-catenin were slightly decreased in *Pcdh20* CKO mice compared to WT mice (Fig. 4g). However, we detected only the T310 and S288 phosphosites of p120-catenin as no commercial antibodies are available for the other phosphosites. The expression of p120-catenin was lower in the colitis group than that in the control group, yet no statistical difference was detected between WT and *Pcdh20* CKO mice with respect to colitis (Fig. 4g). However, phosphosite T310 of p120-catenin was significantly downregulated, while phosphosite S288 was upregulated in *Pcdh20* CKO mice with colitis when compared with WT mice with colitis (Fig. 4g), which was consistent with our phosphoproteomics analysis. Similar results were observed in the Caco-2 and NCM460 cell lines with stable overexpression and knockdown of *PCDH20* during inflammation (Fig. 4h). These findings indicate that PCDH20 deficiency leads to the unzipping of adherens junctions by downregulating β-catenin and dysregulating T310 and S288 of p120-catenin. Together, these data demonstrate that PCDH20 deficiency impairs the epithelial barrier integrity in colitis by unzipping adherens junctions via a dysregulated catenin complex.

### PCDH20 interacts with downstream ER stress chaperone ATF6

**Fig. 5:**
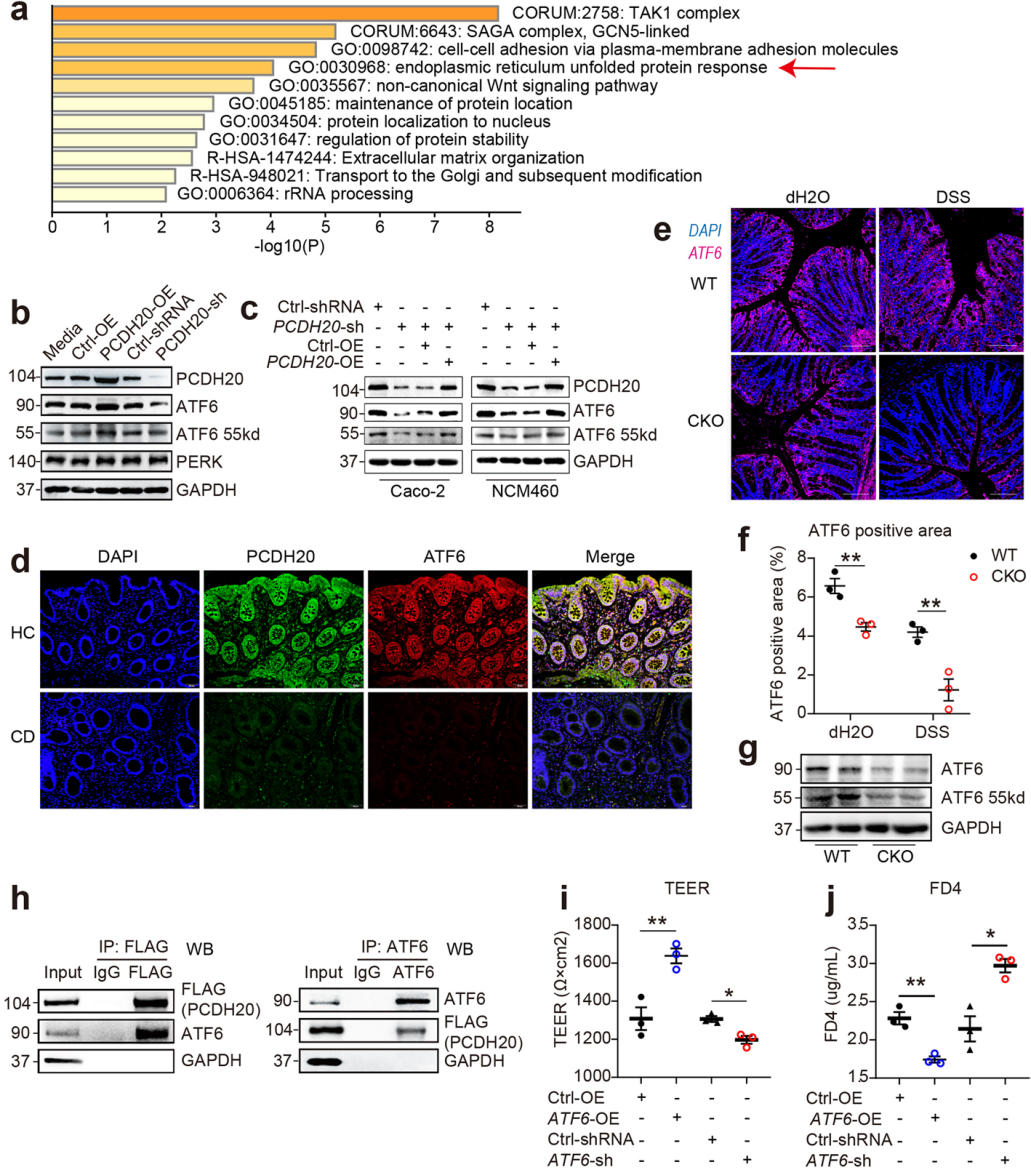
PCDH20 interacted with downstream ATF6. **a** Enriched Ontology clusters heatmap of predicted PCDH20 interactors. **b** Immunoblot of ATF6 expression in Caco-2 cell line with stable *PCDH20* knockdown and overexpression. **c** Immunoblot of ATF6 expression in *PCDH20*-deficient Caco-2 cell line transfected with PCDH20 overexpression plasmid. **d** Decreased ATF6 expression in *PCDH20*-deficient CD patients using immunofluorescence. Magnification × 200, scale bars = 50 μm. Representative images, *n* = 5 for each group. **e–g** Downregulated ATF6 expression in *Pcdh20* CKO mice with DSS-induced colitis compared with WT mice by using immunofluorescence (**e**), semi-quantification of immunofluorescence with Image J software (**f**), and immunoblot (**g**). Magnification × 100, scale bars = 100 μm. Representative images (**e**). Each group *n* = 3. **h** Co-immunoprecipitation of PCDH20 (with flag-tag) and ATF6 in *PCDH20*-overexpressed Caco-2 cell line. **i,j** Paracellular permeability in polarized Caco-2 monolayer transfected with ATF6 overexpression plasmid or ATF6 shRNA. Transepithelial electrical resistance (TEER) (**i**), paracellular permeability assessment with FD4 (**j**). The data in **f**, **i**, **j** were presented as mean ± SEM. * *p* < 0.05 vs. Control, **p < 0.01 vs. Control

To decipher the crosstalk between PCDH20 and the epithelial barrier function in CD, we used BioGRID [22] (Additional file 2: Data S1) (https://thebiogrid.org/) [23] and Metascape (Additional file 3: Data S2) (http://metascape.org/gp/index.html#/main/step1) to predict and analyze the potential interaction targets of PCDH20. We found that the most enriched “biological progress,” the endoplasmic reticulum unfolded protein response (UPR), could play a key role in the crosstalk (Fig. 5a). Two key mediators of UPR, ATF6, and protein kinase RNA-like endoplasmic reticulum kinase (PERK), were predicted as the interaction candidates of PCDH20 (Additional file 3: Data S2). We first assessed the expression of ATF6 and PERK in the Caco-2 cell line with stable overexpression and knockdown of *PCDH20*. Both 90kd and cleaved 55kd forms of ATF6, but not PERK, was expressed in parallel with that of *PCDH20* (Fig. 5b). Meanwhile, transfecting PCDH20 reversed the protein levels (both 90kd and cleaved forms) of ATF6 in two *ATF6*-deficient cell lines (Fig. 5c). Moreover, ATF6 was downregulated in the colonic epithelium of PCDH20-deficient patients with CD (Fig. 5d and Additional file 1: Fig. S14a) and *Pcdh20* CKO mice with colitis (Fig. 5e–g). These data suggest that ATF6 is a downstream protein of PCDH20, and its expression correlates with that of PCDH20. To determine the direct interplay between ATF6 and PCDH20, we constructed a flag-tagged PCDH20 plasmid. Co-immunoprecipitation confirmed the interaction between PCDH20 and ATF6 in both the flag-tagged *PCDH20*-overexpressing Caco-2 cells and Caco-2 cells without any treatment (Fig. 5h, Additional file 1: Fig. S15a). This suggests that PCDH20 interacts with the downstream protein ATF6.

It has been reported that ATF6 is expressed in the ER membrane and nucleus [24], whereas PCDH20 is expressed in the plasma membrane. To further explore the interactive cellular location of PCDH20 and ATF6, we assessed the cellular location of PCDH20 using immunofluorescence and western blotting. Interestingly, in the Caco-2 and NCM460 human colonic cell lines, PCDH20 was located not only in the plasma membrane but also in the cytosol, nucleus, and organelle membranes (Additional file 1: Fig. S14b, c). Co-immunofluorescence staining of the ER marker calnexin and PCDH20 revealed that PCDH20 was also expressed in the ER membrane (Additional file 1: Fig. S14d). These results suggest that PCDH20 could interact with ATF6 in the ER membrane or the nucleus.

Additionally, to determine whether ATF6 regulates epithelial barrier integrity, full-length plasmid and siRNA-ATF6 were constructed and transfected to the monolayers of Caco-2 cells. We then measured TEER and FD4 to assess barrier function. Maximum TEER (Fig. 5i) and minimum FD4 (Fig. 5j) were detected in *ATF6*-overexpressing cells compared with those in control cells, while there was a remarkable reduction in TEER (Fig. 5i) and an increase in FD4 (Fig. 5j) in *ATF6*-deficient cells compared with that in the scramble control. Moreover, ATF6 expression was the most reduced 4 h after inflammatory cytokine treatment in normal human colon epithelial cells, after which, it stably decreased by 18 h of treatment (Additional file 1: Fig. S14e). This reveals that ATF6 plays a crucial role in maintaining the epithelial barrier function during in vitro inflammation, which is consistent with previous in vivo studies that used *Atf6* knockout mice [25]. Thus, PCDH20 deficiency could impair epithelial barrier function by downregulating ATF6 in colitis.

### Selectively activating ATF6 rescues PCDH20-deficiency-associated epithelial barrier impairment in vivo and in vitro

**Fig. 6:**
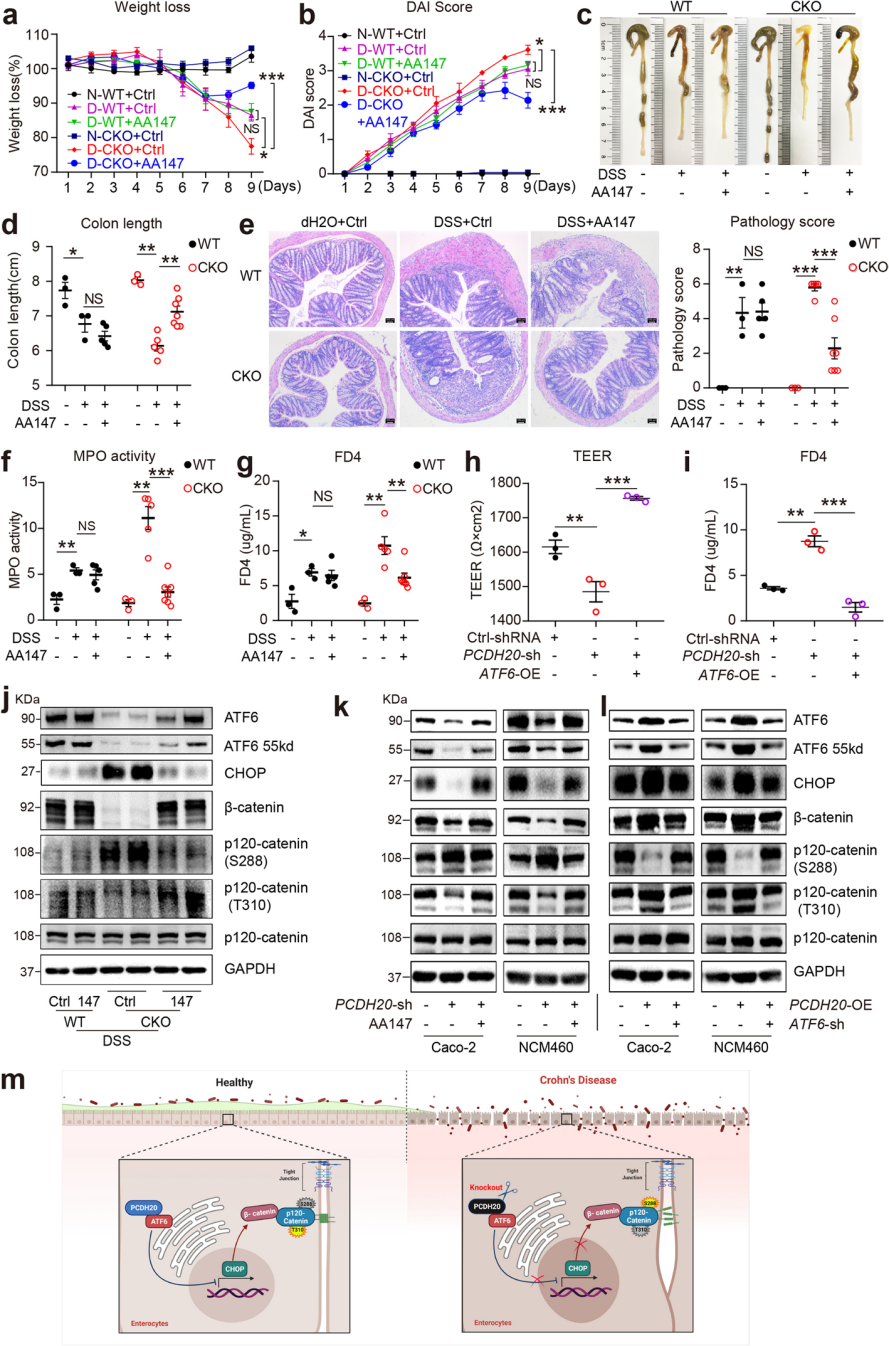
Selective ATF6 activator AA147 rescued PCDH20-deficiency-associated epithelial barrier impairment. **a–f** AA147 rescued severe colitis in *Pcdh20* CKO mice after DSS administration. Weight loss (**a**), disease activity index (**b**), colon length (**c,d**), ulcers, pathology score (**e**), magnification × 100, scale bars = 50 μm), and myeloperoxidase (MPO) activity (**f**). **g** AA147 rescued elevated FD4 level in *Pcdh20* CKO mice with DSS-induced colitis. **h,i** Ameliorated paracellular permeability in *PCDH20*-deficient Caco-2 cells transfected with ATF6 overexpression plasmids along with the treatment of TNFα (2.5 ng/mL), IFN-γ (10 ng/mL). Transepithelial electrical resistance (TEER) (**h**), paracellular permeability assessment with FD4 (**i**). **j** Immunoblots of CHOP and adherens junctions components in *Pcdh20* CKO mice treated with AA147 in DSS colitis group. **k,l** PCDH20 regulated CHOP/β-catenin/p-p120-catenin (S288/T310) by targeting ATF6 in two cell lines treated with TNFα (2.5 ng/mL), IFN-γ (10 ng/mL). **m** Mechanism graph: PCDH20 protects against colitis by tightening adherens junctions partly through the ATF6/CHOP/β-catenin/p-p120-catenin axis. The data in **a–i** are presented as mean ± SEM. * *p* < 0.05, **p < 0.01, ***p < 0.001 vs Control

To investigate whether activating ATF6 improves the impaired barrier function caused by PCDH20 deficiency, we intraperitoneally administered selective ATF6 activator AA147 (0.2 mg/ml) to *Pcdh20* CKO mice prior to, and during, DSS treatment (treatment design see Additional file 1: Fig. S16) [26, 27]. Additionally, AA147 could preferentially increase the expression of ATF6 with a synchronous pattern of both 90kd and cleaved fragments. Treatment with AA147 ameliorated DSS-induced colitis in *Pcdh20* CKO mice, characterized by less weight loss (Fig. 6a), lower DAI (Fig. 6b), longer colon length (Fig. 6c, d), lower levels of pathological scores (Fig. 6e), and lower MPO activity in colonic tissue (Fig. 6f) than those in PBS-treated *Pcdh20* CKO mice with colitis. We also detected significantly decreased intestinal permeability in AA147-treated *Pcdh20* CKO colitis mice compared with those treated with PBS (Fig. 6g). Given the intestinal morphology and disorder of proliferating cells in *Pcdh20* CKO mice, AA147 administration did not restore morphology and proliferation defect in the absence of DSS treatment (Additional file 1: Fig. S17).

Moreover, transfection of the ATF6 plasmid also reversed the decrease in TEER (Fig. 6h) and increase in FD4 (Fig. 6i) in *PCDH20*-deficient cells compared with those in *PCDH20*-deficient cells transfected with the control plasmid. To further investigate the biological functional role of the association between PCDH20 and ATF6, rescue experiments were conducted. Transfection of the ATF6 plasmid at least partly reversed the decrease in TEER and the increase in FD4 in *PCDH20*-deficient cells compared with those transfected with control plasmid (Additional file 1: Fig. S15b-c). While PCDH20 overexpression could completely rescue the decrease in TEER and increase in FD4 in *PCDH20*-deficient cells by reviving ATF6 (Additional file 1: Fig. S15b-d). These data demonstrated that PCDH20 deficiency impairs the epithelial barrier integrity by downregulating ATF6.

### PCDH20 deficiency impairs epithelial barrier integrity by inducing the ATF6/CHOP/β-catenin/p-p120-catenin axis

C/EBP-homologous protein (CHOP) is a reportedly activated downstream chaperone of cleaved ATF6 during ER stress [25, 28] and a suppressor of the β-catenin pathway [29, 30]. Therefore, we hypothesized that PCDH20 deficiency impairs epithelial barrier integrity by regulating the ATF6/CHOP/β-catenin/p-p120-catenin axis. To test this hypothesis, we delineated the regulation of ATF6 on CHOP/β-catenin/p-p120-catenin in the setting of PCDH20 deficiency in vitro and in vivo.

The protein levels of CHOP were increased in *Pcdh20* CKO mice with colitis, while β-catenin was decreased and p-p120-catenin (S288 and T310) was reversed (Fig. 6j). We observed similar results in *PCDH20*-deficient cell lines (Fig. 6k) and the opposite results in the *PCDH20*-overexpressing cells (Fig. 6l). Nevertheless, the expression of the CHOP/β-catenin/p-p120-catenin was partly rescued by cleaved ATF6 activated via AA147 administration in the *Pcdh20* CKO mice (Fig. 6j) and the *PCDH20*-deficient cell lines (Fig. 6k) during colitis. Moreover, elevated *CHOP* mRNA level in the *PCDH20*-deficient cell line was partly reversed by transfection with the ATF6 plasmid (Additional file 1: Fig. S14f). Further, transfection of the ATF6 plasmid could partly reverse the expression of CHOP/β-catenin/p-p120-catenin axis in *PCDH20*-deficient cells compared with those transfected with control plasmid (Additional file 1: Fig. S15d). Whereas PCDH20 overexpression could completely rescue the defect of CHOP/β-catenin/p-p120-catenin axis in *PCDH20*-deficient cells by interacting with ATF6 (Additional file 1: Fig. S15d). These data demonstrate that PCDH20 deficiency impairs epithelial barrier integrity by partly regulating the ATF6/CHOP/β-catenin/p-p120-catenin axis in Crohn’s disease (Fig. 6m).

## Discussion

To the best of our knowledge, our findings provide the first evidence that PCDH20 plays an indispensable role in intestinal epithelial homeostasis. Epithelial *PCDH20* deletion causes defects in intestinal epithelial proliferation, characterized by shortened crypts, aberrant growth of intestinal organoids, and decreased cell proliferation within the bottom of the crypt. PCDH20 ablation also disrupts intestinal epithelial differentiation, characterized by a decreased number of goblet cells and increased number of enterocytes with shortened and twisted microvilli. Actin-based microvilli increase the area of uptake surface area and capacity for solution absorption. Intermicrovillar links in the brush border, known as the densely packed array of microvilli, are assembled by protocadherin-24 and mucin-like protocadherin [31]. Moreover, the morphological changes of the microvilli in *Pcdh20* CKO mice were consistent with the decreased expression of PCDH20 located in the brush border of CD patients. Additionally, altered epithelial differentiation combined with aberrant microvilli, which are critical for solution absorption, could further promote changes in stool consistency [32, 33]. However, unformed and soft stool was rare in the *Pcdh20* CKO mice without intestinal inflammation. One possible explanation is that the increase in uptake, caused by the increased proportion of enterocytes, compensated for the shrinking surface due to reduced microvilli blunting. Thus, the increased proportion of enterocytes could be a consequence of aberrant microvilli caused by the deletion of *PCDH20*.

Notably, the observed shortened microvilli due to *PCDH20* depletion was similar to that of the phenotype of CD patients (decreased microvilli length) [34], suggesting that *PCDH20* deficiency may be a key factor driving the physical defects observed in CD pathogenesis. We also showed that PCDH20 expression was negatively associated with CD severity in patients and in mice with experimental colitis. PCDH20 ablation impaired the intestinal barrier integrity, thus enhancing sensitivity to colitis. Intestinal barrier integrity largely depends on the apical junction complex, which consists of tight junctions, adherens junctions, and apical-to-basal desmosomes [3, 5]. In the setting of *PCDH20* deletion, the increased proportion of enterocytes along with the loosened cell–cell adherens junctions, but not tight junctions, were the predominant causes of the barrier defect in colitis. Nevertheless, reduced secretion of mucin due to decreased number of goblet cells could cause micro dysbiosis in *Pcdh20* CKO mice. Lachnospira, Ruminococcus, Unclassified Clostridium, Clostridium, and Unclassified Firmicutes contributed the most to the dysbiosis after PCDH20 deletion, while Ruminococcus and clostridia were associated with CD [35, 36]. The dysbiosis might lead to mucosal inflammation, which may also contribute to the barrier impairment in *Pcdh20* CKO mice. Collectively, our findings define a critical protective role for PCDH20 in CD development through regulating epithelial barrier integrity.

Additionally, we found that stenosis and perforation due to PCDH20 downregulation were more common in CD patients, while stenosis and perforation were apparent in *Pcdh20* CKO mice with TNBS-induced colitis. Loosened adherens junctions following colitis caused leakage of chemicals and micro pathogens into the lamina propria, which acted as positive feedback for intestinal inflammation and severe histopathological responses, including coagulative necrosis in the epithelium, giant ulcers, severe edema in the lamina propria, and stenosis and perforation in the colon. While stenosis and perforation were both common in *Pcdh20* CKO mice with TNBS colitis, not all patients with CD progress to perforation under natural disease development. Stenosis-associated gene variants in myofibroblasts in the gut could be responsible for the observed heterogeneity in CD patients. Moreover, it has been reported that decreased microvilli length could contribute to the chronic and progressive disease course in patients with CD [34], which is consistent with our findings that a higher CDAI was associated with *PCDH20* deficiency in CD patients and that *PCDH20* ablation in mice caused aberrant microvilli. Further study on PCDH20 combined with stenosis-associated gene variants as histological markers in predicting disease behavior and in guiding treatment strategy for CD patients is warranted.

Recent studies have shown accumulating evidence of PCDH family in cell signaling [37, 38]. Knocking out genes that directly regulate adherens junctions, like *Ndrg2*, *Muc2*, and *Ctnnd1*, causes loosened epithelial adherens junctions without chemical treatment [7, 37, 39]. The abnormity of adherens junctions could even be observed as early as at birth [7]. Nevertheless, we found the gap of adherens junctions only appeared at the late stage of colitis in Pcdh20 CKO mice, suggesting that PCDH20 regulates adherens junctions by inflammation-triggered signaling pathway. Further studies show that PCDH20 interacts with downstream ER stress chaperone ATF6. Moreover, we unveiled that ATF6 is essential for paracellular permeability in vitro during colitis. Similar to our findings, *Atf6*-knockout mice exhibited increased susceptibility to experimental colitis, characterized by a larger ulceration area and elevated expression of CHOP [25], which has been reported as a major apoptotic factor in an UPR [40] and an aggravating factor in colitis [41]. Moreover, mutations in the ATF6 activator, S1P, disrupt ATF6 expression and the UPR, thus, increasing the sensitivity to colitis in mice [42]. P58^*IPK*^, as the downstream transcriptional activation ER chaperone gene of ATF6, was essential to maintaining the amount of goblet cells, decreasing proinflammatory cells, and relieving mucosal impairment under DSS treatment in mice [25]. Further, the bacteria invasion caused by mitochondria dysfunction could be reversed by promoting ATF6–DAPK1 signaling, thus reducing potential intestinal inflammation [43]. Yet, a recent study has revealed that the stable augmented expression of *ATF6* in *Atg16l1* (autophagy gene) knockout mice promotes inflammatory signals in intestinal epithelial cells upon ER stress [44]. Considering that ER stress in secretory cells of intestine could be relieved by eliminating misfolded proteins [45], we postulated that ATF6 upregulation could be an incomplete compensation mechanism for the accumulation of unfolded or misfolded proteins in an autophagy-deficient setting. Overall, ATF6 acts as an important mediator in inflammatory bowel disease pathogenesis connecting the complicated networks of an UPR, ER stress, and autophagy.

AA147 is a newly identified small molecule with capability of selectively activating ATF6 [26]. Recent studies have shown that AA147 plays key theopathetic roles in endothelial barrier function [46], mesodermal differentiation [27], post-cardiac arrest brain injury [47], glutamate-induced oxidative toxicity [48], and liver ischemia/reperfusion injury [49]. We found that AA147 administration reverses elevated CHOP expression, barrier defects, and colitis susceptibility in *Pcdh20* CKO mice with colitis, by selectively increasing both cleaved and 90kd fragments of ATF6. Similar results could be observed in vitro under inflammation. Yet, without inflammation, AA147 did not affect defects of intestinal morphology and Ki67 + cells in *Pcdh20* CKO mice. Collectively, our study demonstrated that PCDH20 played a homeostatic role in intestinal biology, which was independent of ATF6. Moreover, we found that PCDH20 deficiency disrupted the intestinal barrier function by suppressing the ATF6-mediatied ER stress pathway in colitis, which indicated that PCDH20 reduction played a significant role in intestinal inflammation dependent on ATF6. However, short-term ATF6 activation might not be sufficient to rescue defects established during early life of *Pcdh20* CKO mice.

## Conclusions

This study provides novel insights into the mechanism of PCDH20-dependent intestinal barrier function and intestinal homeostasis in CD. We demonstrate that *PCDH20* deficiency unzips the adherens junctions between enterocytes by suppressing the ATF6/CHOP/β-catenin/p120-catenin pathway, thereby disrupting barrier integrity and enhancing sensitivity to experimental colitis. This could be attributed to the stenosis or perforation phenotype observed in patients with CD. Hence, PCDH20 deficiency could be considered a useful prognostic predictor for progressing disease behavior. Moreover, PCDH20 upregulation or the selective activation of ATF6 could be a novel therapeutic strategy to increase the epithelial integrity for CD treatment.

## Methods

The experimental methods comply with the Helsinki Declaration.

### Human tissue collection

Colon biopsy samples were collected from 43 patients with CD and 42 healthy individuals who underwent colonoscopy (clinical characteristics see Additional file 1: Table S1). The Crohn’s disease activity index (CDAI) was evaluated upon initial sample collection.

This study was approved by the Clinical Research Ethics Committees of The First Affiliated Hospital of Sun Yat-sen University (the approval number [2017]072). Informed consent was also obtained from all patients before biopsies were collected.

### Cell culture

Human colonic epithelial Caco-2 cell line derived from colorectal adenocarcinoma was purchased from the American Type Culture Collection (ATCC, Rockville, MD, USA) and was maintained with 20% fetal bovine serum (Gibco) in Eagle’s Minimum Essential Medium (ATCC). Human epithelial NCM460 cell line derived from normal colon was purchased from InCell (Texas, USA) and was maintained with 10% FBS in M3 Medium (InCell). Cells were cultured at 37 °C incubator with 5% CO_2_. Low passages of cell line were used in this study. Both cell lines were authenticated by short tandem repeat fingerprinting and were tested for mycoplasma before use.

### Intestinal epithelial Pcdh20 conditional knockout (CKO) mice

Intestinal epithelial *Pcdh20* conditional knockout (CKO) mice with C576BL/6j background were generated using the Cre/loxP system by the Nanjing Biomedical Research Institute of Nanjing University. Briefly, to generate mice carrying loxP-flanked alleles of *Pcdh20* (*Pcdh20*^fl/fl^), the exon 3 of *Pcdh20-001* gene was targeted, and CRISPR/Cas9 system was conducted to mediate loxP site insertion. Cas9 mRNA, sgRNA, and donor with loxP sites were co-injected into mice zygotes. sgRNA directed Cas9 endonuclease cleavage in intron 2–3 and downstream of 3′UTR, and created double-strand break. By homologous recombination, LoxP sites was inserted to flank exon 3 of *Pcdh20. Pcdh20*^fl/fl^ mice were crossed with Villin-Cre mice (The Jackson Laboratory, USA) to generate Villin-Cre *Pcdh20*^fl/fl^ (*Pcdh20* CKO) and wild-type (WT) mice. All mice were raised under specific pathogen-free conditions. The animal studies were approved by the Animal Research Ethics Committees of The First Affiliated Hospital of Sun Yat-sen University (the approval number [2017]061).

### Establishment of DSS- and TNBS-induced colitis

Briefly [50], dextran sulfate sodium (DSS; MP Biomedicals, USA) was dissolved in drinking water to obtain 2% DSS solution, and the mice were provided fresh DSS solution to drink for 7 days. Mice in the control group were given drinking water alone. All mice were sacrificed on day 10.

Next, 1% 2,4,6-trinitrobenzenesulfonic acid solution (TNBS; Sigma-Aldrich USA) was administered to the mice to induce pre-sensitization. After 7 days, the mice were fasted for 18 h, anesthetized with isoflurane, and administered intrarectally with 150 μL 2.5% TNBS in 50% ethanol. Mice in the control group were administered 50% ethanol alone. Due to the high death rate of *Pcdh20* CKO mice in the TNBS group (Additional file 1: Fig. S18), the mice were sacrificed on day 3.

Disease activity index (DAI) [50], myeloperoxidase (MPO) activity, and histopathological analysis [50] can be found in the Supplementary Methods (Additional file 4: Supplementary Methods).

### In vivo* intestinal permeability detection*

Fluorescein isothiocyanate-dextran 4 kDa (FD4; Sigma-Aldrich, USA) was used to determine intestinal paracellular permeability. In brief [51], mice subjected to fasting for 10 h were weighed before gavage. The mice were orally administered with FD4 in PBS (22 mg/mL) at corresponding to a volume of 22 mL/kg body weight. After 4 h, the blood sample was collected and centrifuged at 3000 rpm and 4 °C for 20 min. The FD4 level in the serum was detected using a spectrophotometer (excitation/emission wavelength: 485/530 nm, SpectraMax M5, Molecular Devices, USA) and calculated using the standard curve of FD4.

### Transepithelial electrical resistance (TEER) and paracellular permeability assessment

Caco-2 cells stably transfected with different vectors (details see Additional file 4: Supplementary Methods) were seeded at 10,000 cells/cm^2^ on transwell filters with 0.4-μm polyester membrane (Corning) and allowed to grow for 20 days to develop a monolayer. The cells were cultured with complete medium containing 2.5 ng/mL tumor necrosis factor-alpha (TNFα; PeproTech) and 10 ng/mL interferon-gamma (IFN-γ; PeproTech) [52]. The TEER was measured on day 21. The resistance value (ohms, Ω) was detected by Millicell ERS-2 Voltohmmeter (Millipore, Darmstadt, Germany), and the TEER was calculated using the following formula:

$$TEER\left(\Omega \bullet {cm}^{2}\right)=\left(\mathrm{measured ohms}-\mathrm{blank insert ohms}\right)(\Omega )\times \mathrm{filter area }({\mathrm{cm}}^{2})$$

Paracellular permeability was assessed by FD4 (Sigma-Aldrich) flux through the caco-2 cell monolayer, as previously described [53]. Opti-MEM medium (Gibco) supplemented with FD4 (2 mg/mL) was added to the upper compartment on day 21, as described in the TEER procedure. Next, Opti-MEM medium was added to the lower compartment and incubated for 4 h at 37 °C; 200 μL of media was collected from the lower compartment to detect FD4 fluorescence intensity using a spectrophotometer (SpectraMax M5, Molecular Devices, CA, USA).

### Organoid isolation, culture, and observation

Intestinal organoids were isolated and cultured as previously described [54]. In brief, after three washes with PBS (Gibco), intestinal tissues were minced and incubated with 2 mM ethylenediaminetetraacetic acid (Gibco) for 20 min at 37 °C under rotation. Afterward, the samples were vigorously shaken and filtered with a 100-μm strainer to release single crypts. The isolated crypts were centrifugated 2 times with 200 × *g* for 5 min, embedded in Matrigel (Corning Lifescience), and cultured with mouse Organoid Growth Medium (STEMCELL). Media was replaced every 2 days. Images of organoids were captured by an inverted microscope (DMi8, Leica, Germany).

### Co-immunoprecipitation

Co-immunoprecipitation was performed using the Direct Magnetic IP/Co-IP Kit (Thermo Scientific) following the manufacturer’s protocols. Primary antibodies (5 μg/reaction) against Flag (#14,793, CST), ATF6 (sc-166659, Sigma-Aldrich), rabbit IgG (#3900, CST), and mouse IgG1 kappa (14–4714-82, eBioscience) were used respectively to couple to N-hydroxysuccinimide-activated magnetic beads (25 μL/reaction) for 30 min at room temperature. The cells were lysed and extracted using lysis buffer. The protein concentration was then estimated using the Bicinchoninic acid Protein Assay Kit (88,828, Thermo Scientific). Lysate solution with 750 μg of proteins was added to the antibody-coupled magnetic beads and incubated overnight at 4 °C on a shaker (88,881,002, Thermo Scientific) for antigen immunoprecipitation. The immunoprecipitation complex was eluted using a magnetic stand (21,359, Thermo Scientific), and the eluted proteins were electrophoresed and analyzed by western blotting.

### TMT-labeled quantitative proteomics and phosphoproteomics quantification analysis

Colon samples from the *Pcdh20* CKO and WT mice (*n* = 3/group) were frozen using liquid nitrogen and ground into cell powder. The powder was then lysed using lysis buffer (8 M urea and 1% protease inhibitor cocktail) and sonicated on ice with a high-intensity ultrasonic processor (Scientz). Proteins were digested with trypsin (Promega) to obtain peptides, which were desalted, vacuum-dried, and processed with TMT labeling according to the TMT kit (Thermo) manufacturer’s protocol. The tryptic peptides were fractionated by high pH reverse-phase high-pressure liquid chromatography (HPLC) using a Thermo Betasil C18 column for further liquid chromatography with tandem mass spectrometry (LC–MS/MS) analysis.

To conduct phosphoproteomic analysis, the sample powder was lysed using lysis buffer (8 M urea, 1% protease inhibitor cocktail, and 1% phosphatase inhibitor cocktail). Protein sonication, digestion, TMT labeling, and HPLC fractionation were performed, as described above. For biomaterial-based PTM enrichment, phosphopeptides were enriched using an IMAC microsphere suspension in loading buffer (50% acetonitrile/6% trifluoroacetic acid), eluted, desalted, and lyophilized for LC–MS/MS analysis.

All LC–MS/MS and bioinformatics analyses were performed by PTM Biolabs Inc (Hangzhou, China). To ensure high confidence, the standard of localization probability (> 0.75) was used to filter the data, and the quantified values of the filtered phosphorylation modification sites were normalized to those of proteins. Data analysis was conducted with Maxquant (v1.5.2.8).

### Statistical analysis

The in vitro experiments were repeated thrice to generate three biological replicates. The data were presented as mean ± SEM and were analyzed with GraphPad Prism 8 software (GraphPad Software Inc., USA). Student’s *t* test or Welch’s *t* test was used to analyze the differences between two groups, whereas one-way ANOVA was conducted to compare more than two groups. Non-parametric statistical analysis was performed for datasets with skewed distribution. *P* values < 0.05 were considered statistically significant.

## Supplementary Information

**Additional file 1: Fig S1.** The mRNA expression heatmap of cadherin family in colonic epithelium of CD patients. **Fig S2.** PCDH20 expression in inflamed tissue of CD patients and successful construction of DSS and TNBS colitis. a Decreased expression of PCDH20 in inflamed tissue of CD patients. b-c Successful construction of TNBS colitis, greater weight loss, shortened colon length. d-g Successful construction of DSS colitis, greater weight loss, higher disease activity index, shortened colon length, and higher myeloperoxidaseactivity. Each group *n* > 3. * *p*<0.05, **p<0.01 vs. Control, ***p<0.001 vs Control. **Fig S3.** Baseline of *Pcdh20* CKO mice. a-b The expression of PCDH20 in *Pcdh20* CKO mice, using qRT-PCRand immunoblot. c Shortened villi in ileum of *Pcdh20* CKO mice. d Tight junction of colonic epithelial cells in CKO mice. Each group *n* =3. ***p<0.001 vs WT. **Fig. S4.** Establishment of colonic cell lines with stable PCDH20 overexpression and knockdown.a PCDH20 mRNA expression in different colonic cell lines. b GFP expression in transfected cell lines detected by fluoresce microscope. c-d PCDH20 expression in PCDH20 overexpression and knockdown colonic cell lines, using using qRT-PCRand immunoblot. * *p*<0.05 vs. Control. **Fig. S5.** Quantificationof differentiated cells in the colon epithelium of *Pcdh20* CKO mice. a The number and percentage of goblet cells in colon crypt. b The IOD value of FABP1+ enterocytes. c The number and percentage of enteroendocrine cells in colon crypt. * *p*<0.05, **p<0.01, ***p<0.001 vs WT. Fig S6 Epithelial differentiation of Pcdh20 CKO mice. Representative images of differentiated cells in the ileum epithelium of Pcdh20 CKO mice. Magnification ×200. Each group *n* = 3. **Fig S7.** The effect of knocking out PCDH20 on gut microbiota. a At the phylum level, the effect of intestinal-specific knockout of PCDH20 on the gut microbiota. b At the genus level, the effect of intestinal-specific knockout of PCDH20 on gut microbiota. c At the species level, the effect of intestinal-specific knockout of PCDH20 on gut microbiota. d At the species level, the heat map of the differential gut microbes after intestinal-specific knockout of PCDH20. e Comparison of alpha diversity between groups. f Principal coordinatesanalysis based on Bray-Curtis distance at the phylum level. Each group n = 3. **Fig S8.** The differential gut microbiota of PCDH20 on gut microbiota. a Distribution of LDA values for differential species.t. b Evolutionary clade diagram of different species. c At the genus level, clustering heatmap based on differential species. **Fig. S9.** Statistical plot of KEGG-annotated metagenomic gene number. **Fig. S10.** Intestinal permeability, cellular ultrastructure in Pcdh20 CKO mice with TNBS-induced colitis. a-c More severe colitis in Pcdh20 CKO mice after TNBS administration. Weight loss, colon length, myeloperoxidaseactivity. d Increased FD4 level detected in the serum of Pcdh20 CKO mice with TNBS-induced colitis. Each group *n* ≥ 3. * *p*<0.05, **p<0.01 vs. WT. **Fig. S11.** Log2 of TEER in PCDH20 deficient cell line at different time points. **Fig. S12.** Proteomics and phosphoproteomics analysis on Pcdh20 CKO mice with DSS colitis. a-c Quality control analysis, including principal component analysis, relative standard deviationand Pearson Correlation. Proteomics, phosphoproteomics. d Volcano plots of proteins with fold change over 1.3 in proteomicsand phosphosites with fold change over 1.5 in phosphoproteomics. e Gene ontologyterms of the top biological process regulated in phosphoproteomics of colon of Pcdh20 CKO mice with DSS-induced colitis. f The network of proteins involved in adheren junctions analysed by Cytoscape using data from phosphoproteomics. Each group *n* = 3. **Fig. S13. **Adherens junctions in the colon epithelium of Pcdh20 CKO mice with DSS-induced colitis. a Adherens junctions observed by transmission electron microscope. Magnification ×3000, scale bars = 2μm. b Microvilli length of PCDH20 CKO mice with colitis at different time points. Fig. S14. Cellular location of PCDH20 and expression of ATF6 and its downstream CHOP. a ATF6 expression in the colonic epithelium of PCDH20-deficient CD patients. b-c Expression and location of PCDH20 in different cell components, using immunoblotand immunofluorescence. d Co-localization analysis of PCDH20 and ER marker Calnexin. e ATF6 expression in NCM460 cells with TNFαand IFN-γtreatment. f Decreased CHOP mRNA level in PCDH20-deficient cell line transfected with ATF6 plasmid. ***p<0.001 vs Control. **Fig. S15.** The interaction and function of PCDH20 and ATF6. a Co-immunoprecipitation of PCDH20 and ATF6 in Caco-2 cell line. b-c Ameliorated paracellular permeability in PCDH20-deficient Caco-2 cells transfected with different plasmid under inflammatory stimulation. Transepithelial electrical resistance, paracellular permeability assessment with FD4. d Rescued immunoblots of CHOP/β-catenin in two PCDH20-deficient cell lines transfected with different plasmid under inflammatory stimulation. **Fig. S16.** The experimental design and timeline of AA147 treatment in mice with DSS colitis. **Fig. S17.** The effect of AA147 on epithelial morphology and proliferation in Pcdh20 CKO mice. PCDH20 on gut microbiota. a Crypt length in colon stained with H&E. Magnification ×100, scale bars = 50μm. b microvilli observed with transmission electron microscope. Magnification ×8000, scale bars = 1μm. c Representative images of ki67 stained with immunohistochemistry in colon mucosa. Magnification ×200, scale bars = 100μm. Each group *n* = 3. The data were presented as mean ± SEM. * *p*<0.05, vs. Ctrl. Intestinal epithelial conditional knockout, CKO. **Fig. S18.** The survival rate of PCDH20 CKO mice with TNBS colitis. WT *n* = 9, CKO *n* = 12, * *p*<0.05. **Table S1.** The clinical characteristics of Crohn’s disease patients and controls selected for RNA-seq. **Table S2.** Clinical characteristics of patients with CD and healthy controls.

**Additional file 2: Data S1** Interactors of PCDH20 downloaded from Biogrid.

**Additional file 3: Data S2** Enrichment of PCDH20 interactors using Metascape.

**Additional file 4.** Supplementary methods.

**Additional file 5.** Peer review history.

## Data Availability

The RNA sequencing data is available at NCBI Gene Expression Omnibus (GEO) [https://www.ncbi.nlm.nih.gov/geo/query/acc.cgi?acc=GSE230113] under the accession number GSE230113 [16] [platform ID: GPL16791; dataset IDs: GSM7187739- GSM7187758]. The published datasets analyzed during the current study are available in the GEO and Database of Genotypes and Phenotypes (dbGaP) repository, https://www.ncbi.nlm.nih.gov/geo/query/acc.cgi?acc=GSE59071 and https://www.ncbi.nlm.nih.gov/projects/gap/cgi-bin/study.cgi?study_id=phs000424.v8.p2 [11, 15]. Re-investigation data of existing dataset, which are openly available in the BioGRID at https://thebiogrid.org/122337/summary/homo-sapiens/pcdh20.html/ [55], are included in the supplementary materials. All microscopy images and uncropped blot images are available at Figshare and can be accessed at (microscopy images: 10.6084/m9.figshare.23267519 [56], uncropped blot images: 10.6084/m9.figshare.23266235 [57]).

## Abbreviations

ATF6: Activating transcription factor 6
CD: Crohn’s disease
CDAI: Crohn’s disease activity index
CHOP: C/EBP-homologous protein
CKO: Intestinal conditional knockout
DAI: Disease activity index
DSS: Dextran sulfate sodium
FD4: FITC-dextran 4 kDa
HPLC: High-pressure liquid chromatography
IFN-γ: Interferon-gamma
LC–MS/MS: Liquid chromatography with tandem mass spectrometry
MPO: Myeloperoxidase
PERK: Protein kinase RNA-like endoplasmic reticulum kinase
PCDH: Protocadherin
PCDH20: Protocadherin 20
TEER: Transepithelial electrical resistance
TEM: Transmission electron microscope
TNBS: 2,4,6-Trinitrobenzenesulfonic acid solution
TNFα: Tumor necrosis factor-alpha
UPR: Unfolded protein response
WT: Wild-type
