# Supplementary material for: Protocadherin 20 maintains intestinal barrier function to protect against Crohn’s disease by targeting ATF6
Source: Genome Biol. 2023 Jul 5;24:159. doi: 10.1186/s13059-023-02991-0 (PMC10320870; doi:10.1186/s13059-023-02991-0)
Supplement: Supplementary file 1 — Additional file 1: Fig S1. The mRNA expression heatmap of cadherin family in colonic epithelium of CD patients. Fig S2. PCDH20 expression in inflamed tissue of CD patients and successful construction of DSS and TNBS colitis. a Decreased expression of PCDH20 in inflamed tissue of CD patients. b-c Successful construction of TNBS colitis, greater weight loss, shortened colon length. d-g Successful construction of DSS colitis, greater weight loss, higher disease activity index, shortened colon length, and higher myeloperoxidaseactivity. Each group n > 3. * p<0.05, **p<0.01 vs. Control, ***p<0.001 vs Control. Fig S3. Baseline of Pcdh20 CKO mice. a-b The expression of PCDH20 in Pcdh20 CKO mice, using qRT-PCRand immunoblot. c Shortened villi in ileum of Pcdh20 CKO mice. d Tight junction of colonic epithelial cells in CKO mice. Each group n =3. ***p<0.001 vs WT. Fig. S4. Establishment of colonic cell lines with stable PCDH20 overexpression and knockdown.a PCDH20 mRNA expression in different colonic cell lines. b GFP expression in transfected cell lines detected by fluoresce microscope. c-d PCDH20 expression in PCDH20 overexpression and knockdown colonic cell lines, using using qRT-PCRand immunoblot. * p<0.05 vs. Control. Fig. S5. Quantificationof differentiated cells in the colon epithelium of Pcdh20 CKO mice. a The number and percentage of goblet cells in colon crypt. b The IOD value of FABP1+ enterocytes. c The number and percentage of enteroendocrine cells in colon crypt. * p<0.05, **p<0.01, ***p<0.001 vs WT. Fig S6 Epithelial differentiation of Pcdh20 CKO mice. Representative images of differentiated cells in the ileum epithelium of Pcdh20 CKO mice. Magnification ×200. Each group n = 3. Fig S7. The effect of knocking out PCDH20 on gut microbiota. a At the phylum level, the effect of intestinal-specific knockout of PCDH20 on the gut microbiota. b At the genus level, the effect of intestinal-specific knockout of PCDH20 on gut microbiota. c At the species level, the effect [file 13059_2023_2991_MOESM1_ESM.docx]

Fig S1.


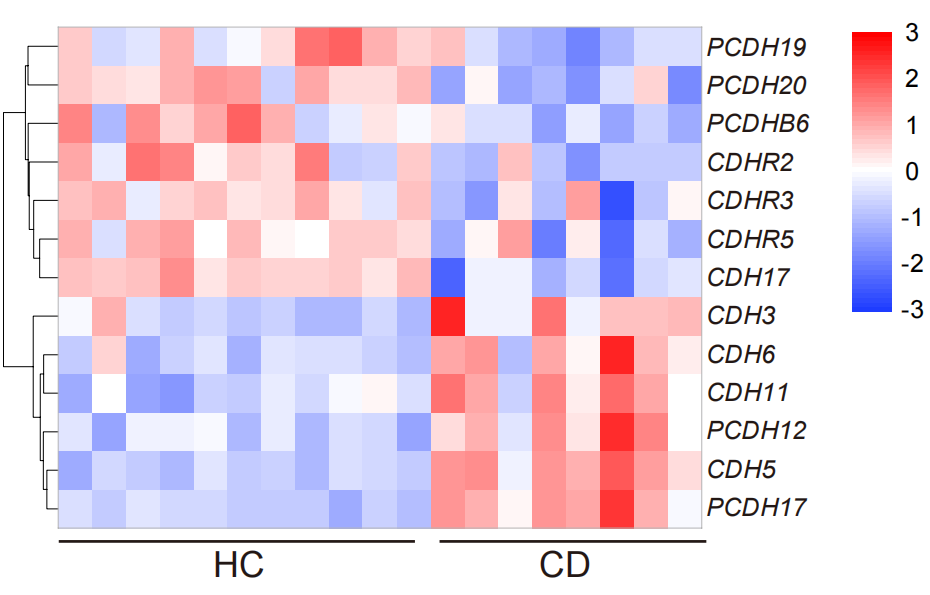


**Fig S1** The mRNA expression heatmap of cadherin family in colonic epithelium of CD patients (GSE59071).

Fig S2.


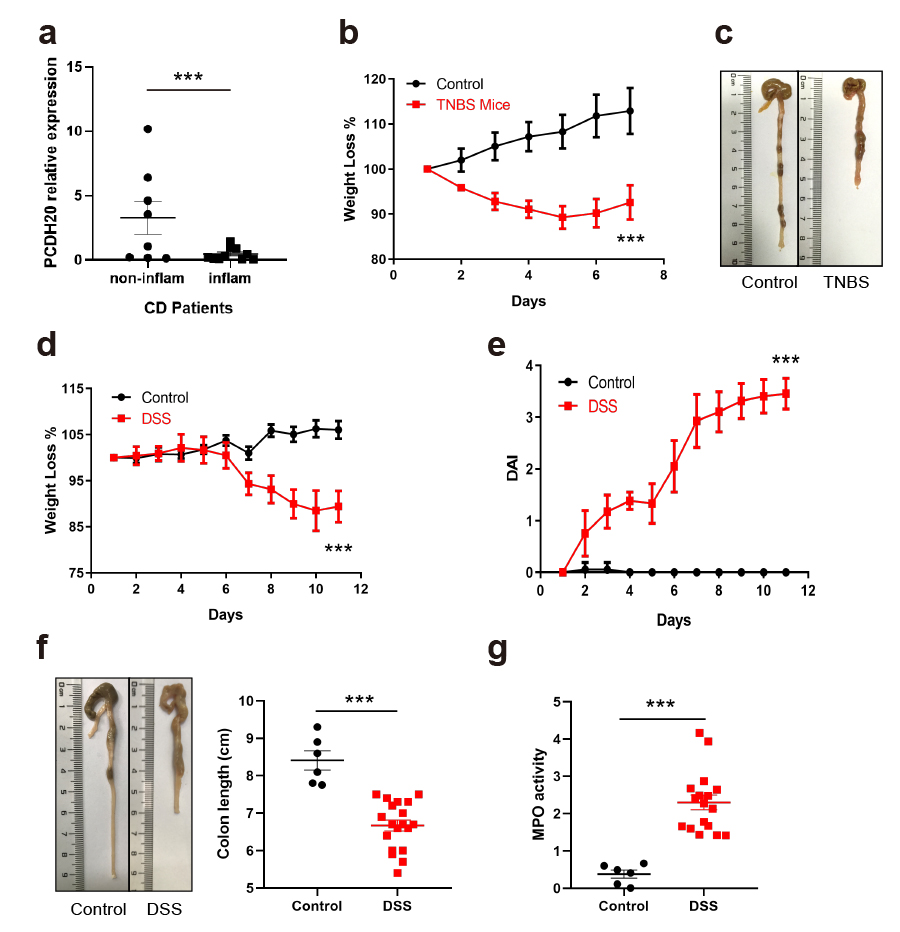


**Fig S2** PCDH20 expression in inflamed tissue of CD patients and successful construction of DSS and TNBS colitis. **a** Decreased expression of PCDH20 in inflamed tissue of CD patients. **b-c** Successful construction of TNBS colitis, greater weight loss (**b**), shortened colon length (**c**). **d-g** Successful construction of DSS colitis, greater weight loss (**d**), higher disease activity index (DAI) (**e**), shortened colon length (**f**), and higher myeloperoxidase (MPO) activity (**g**). Each group n > 3. * p<0.05, **p<0.01 vs. Control, ***p<0.001 vs Control.

Fig S3.


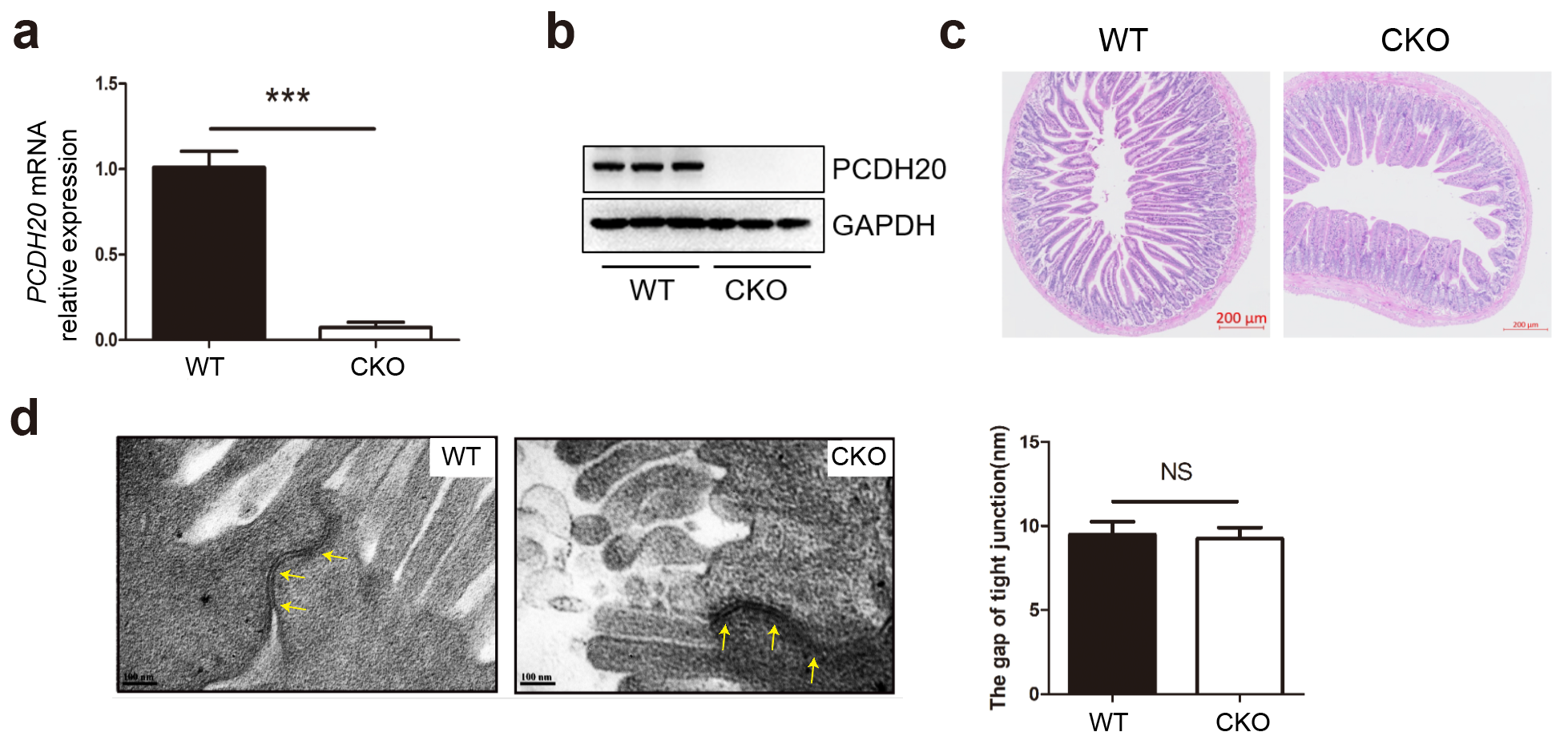


**Fig S3** Baseline of *Pcdh20* CKO mice. **a-b** The expression of PCDH20 in *Pcdh20* CKO mice, using qRT-PCR (**a**) and immunoblot (**b**). **c** Shortened villi in ileum of *Pcdh20* CKO mice. **d** Tight junction of colonic epithelial cells in CKO mice. Each group n =3. ***p<0.001 vs WT.

Fig S4.


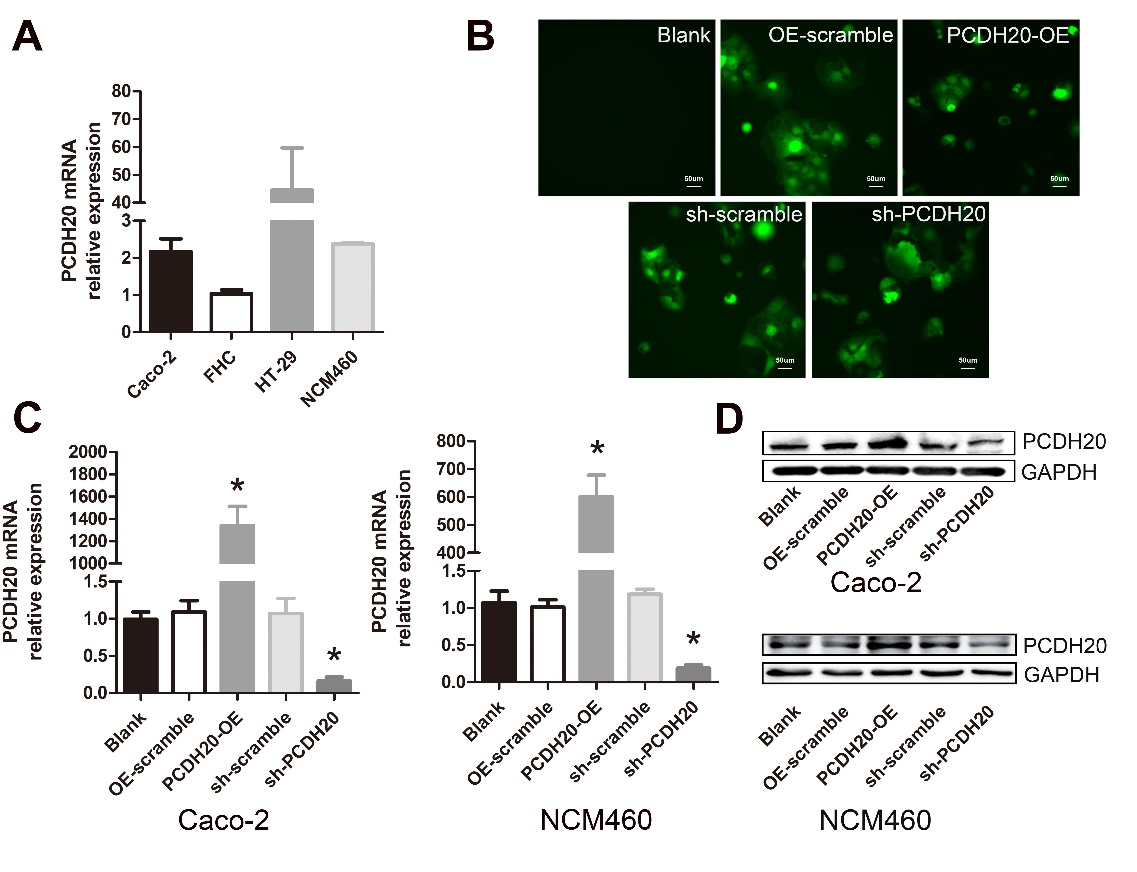


**Fig S4** Establishment of colonic cell lines with stable PCDH20 overexpression and knockdown.

**a** PCDH20 mRNA expression in different colonic cell lines. **b** GFP expression in transfected cell lines detected by fluoresce microscope. **c-d** PCDH20 expression in PCDH20 overexpression and knockdown colonic cell lines, using using qRT-PCR (**c**) and immunoblot (**d**). * p<0.05 vs. Control.

Fig S5.


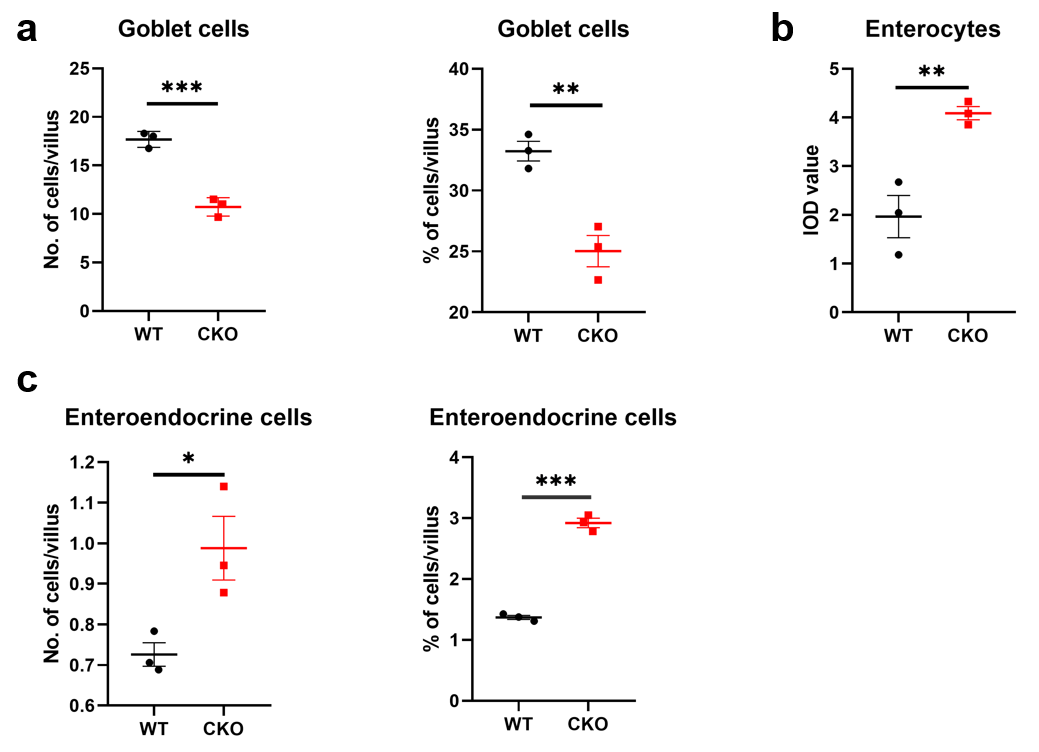


**Fig S5** Quantificationof differentiated cells in the colon epithelium of *Pcdh20* CKO mice. **a** The number and percentage of goblet cells in colon crypt. **b** The IOD value of FABP1+ enterocytes. **c** The number and percentage of enteroendocrine cells in colon crypt.

* p<0.05, **p<0.01, ***p<0.001 vs WT.

Fig S6.


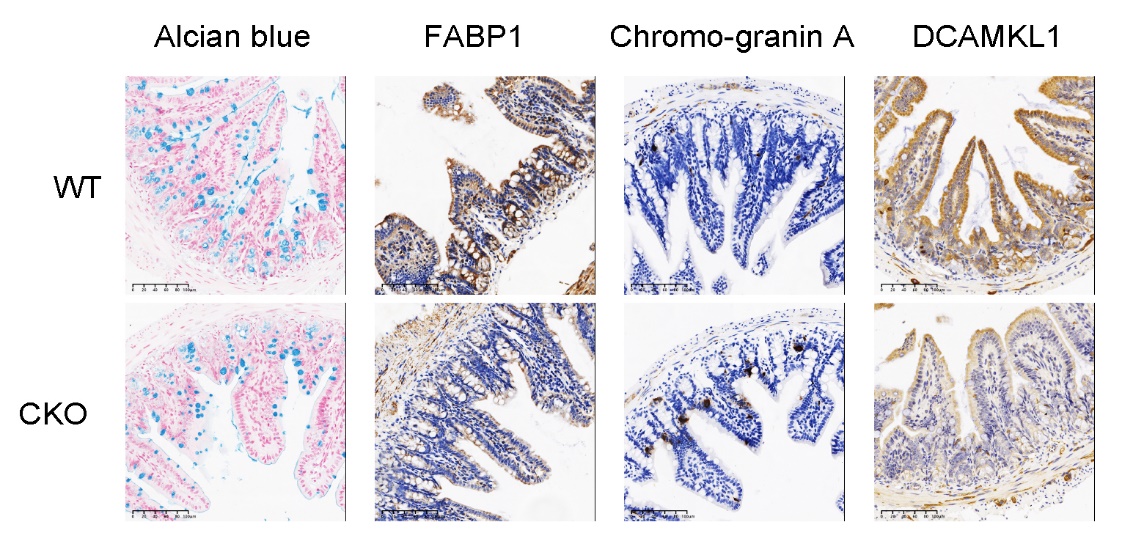


**Fig S6** Epithelial differentiation of Pcdh20 CKO mice. Representative images of differentiated cells in the ileum epithelium of Pcdh20 CKO mice (Alician blue, goblet cells; FABP1, enterocytes; Chromo-graninA, enteroendocrine cells; DCAMK1, Tuft cells). Magnification ×200. Each group n = 3.

Fig S7.


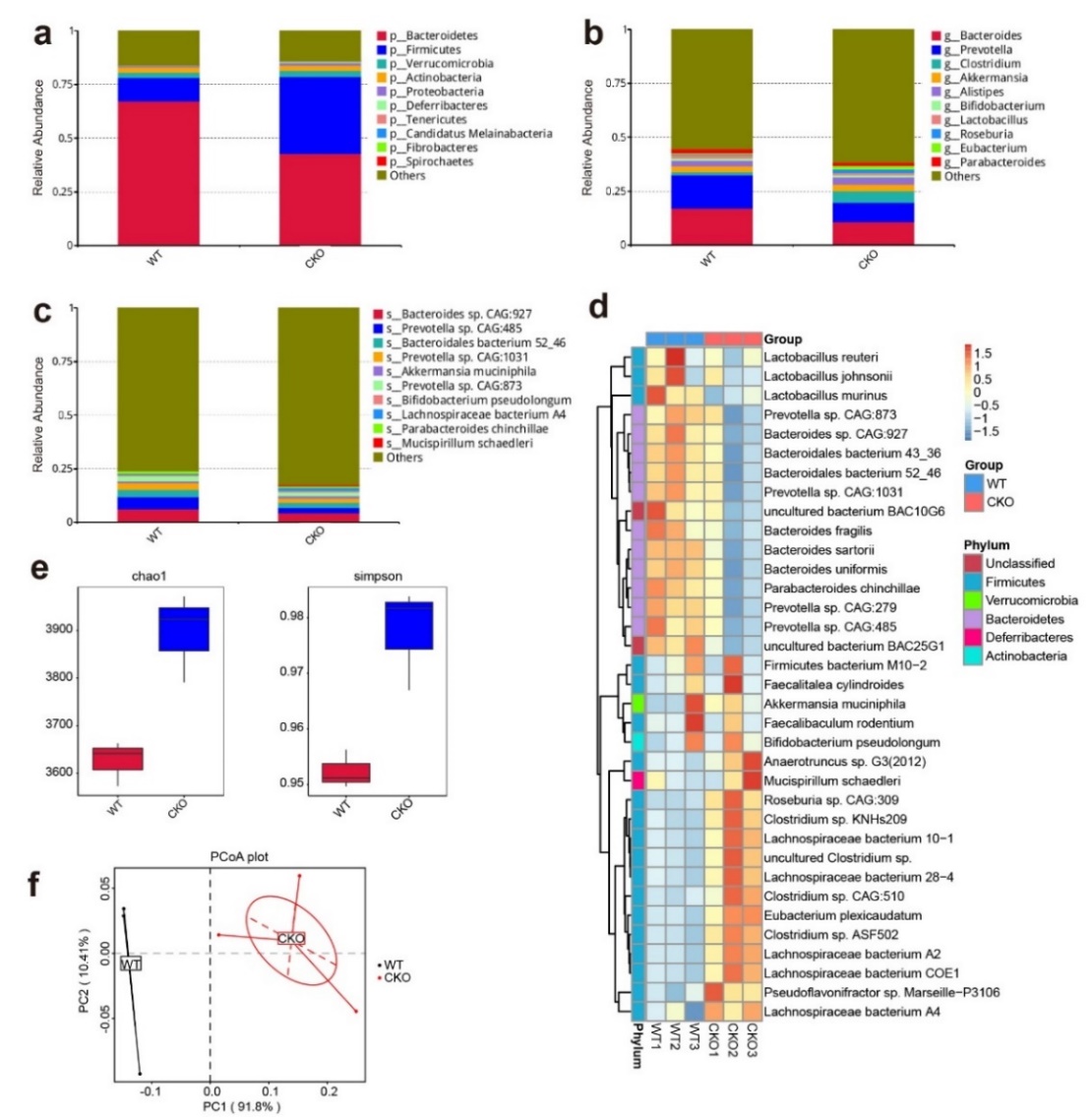


**Fig S7** The effect of knocking out PCDH20 on gut microbiota. **a** At the phylum level, the effect of intestinal-specific knockout of PCDH20 on the gut microbiota. **b** At the genus level, the effect of intestinal-specific knockout of PCDH20 on gut microbiota. **c** At the species level, the effect of intestinal-specific knockout of PCDH20 on gut microbiota. **d** At the species level, the heat map of the differential gut microbes after intestinal-specific knockout of PCDH20. **e** Comparison of alpha diversity between groups (chao1 index and Simpson index). **f** Principal coordinates (PCoA) analysis based on Bray-Curtis distance at the phylum level. Each group n = 3.

Fig S8.


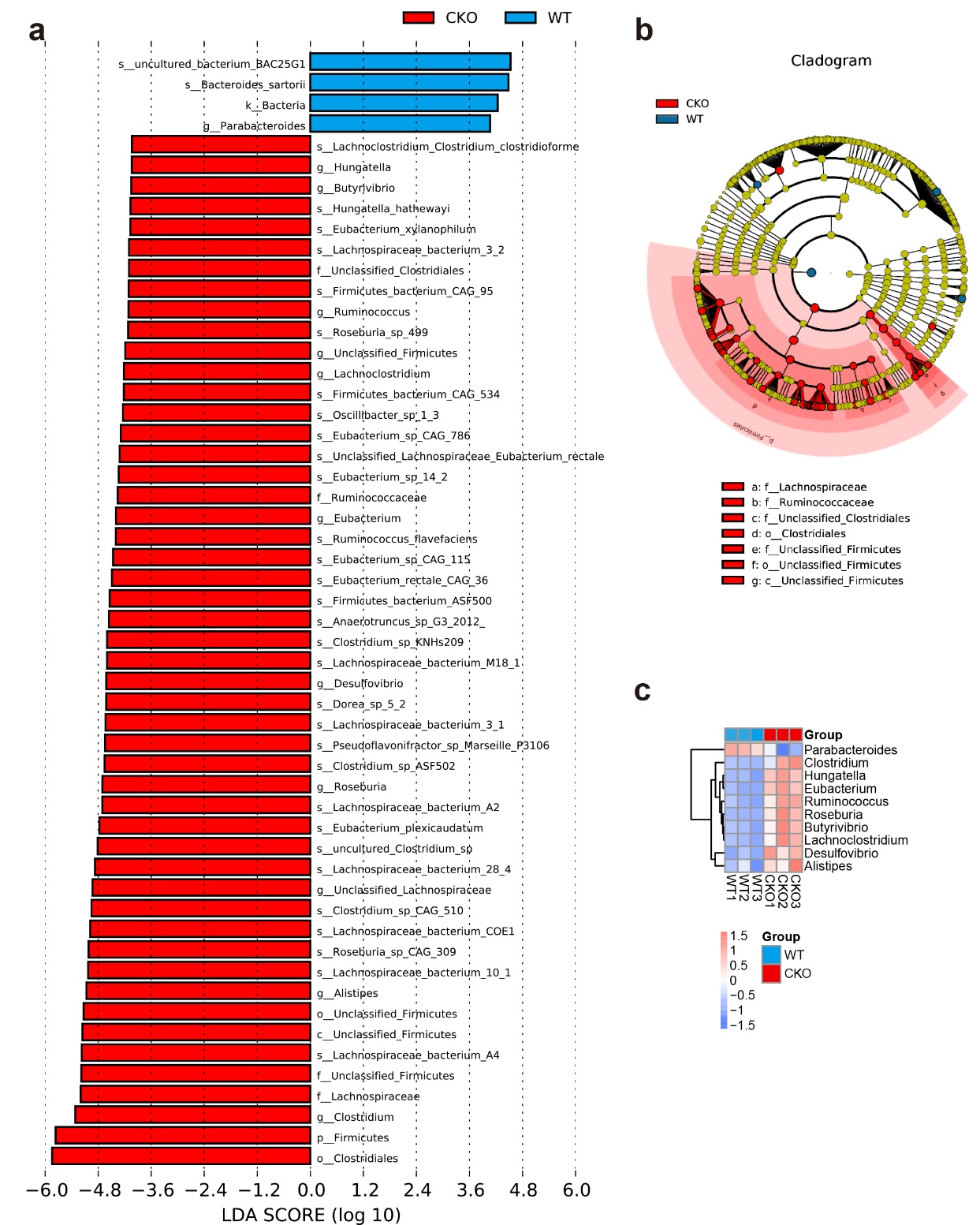


**Fig S8** The differential gut microbiota of PCDH20 on gut microbiota. **a** Distribution of LDA values for differential species.t. **b** Evolutionary clade diagram of different species. **c** At the genus level, clustering heatmap based on differential species.

Fig S9.


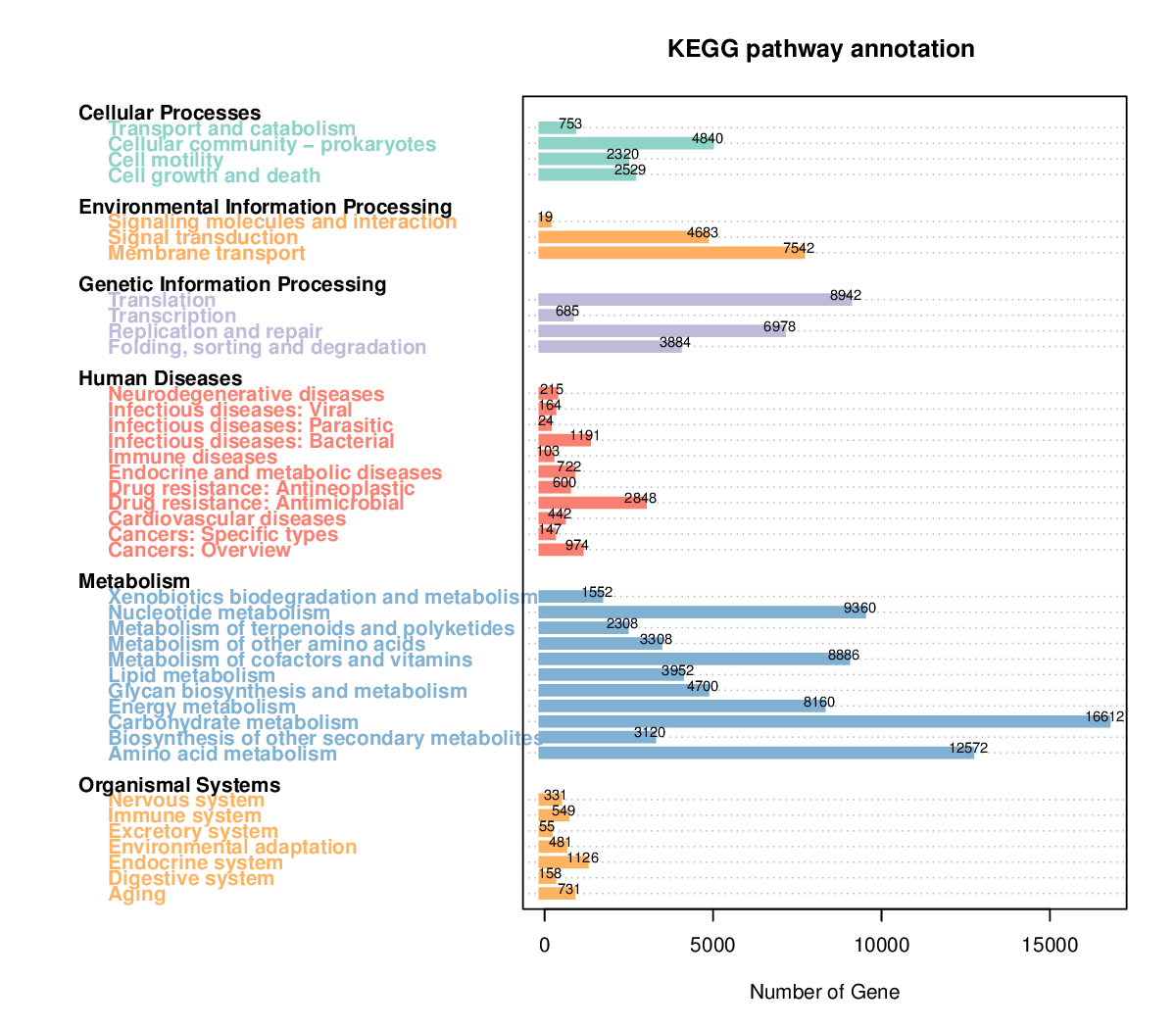


**Fig S9** Statistical plot of KEGG-annotated metagenomic gene number.

Fig S10.


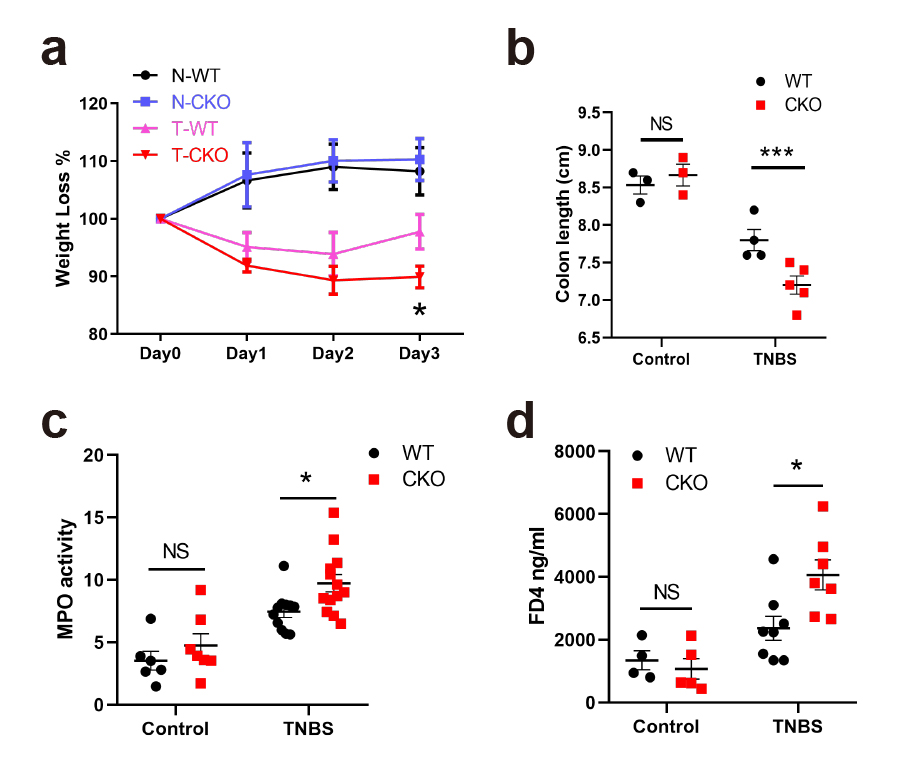


**Fig S10** Intestinal permeability, cellular ultrastructure in Pcdh20 CKO mice with TNBS-induced colitis. **a-c** More severe colitis in Pcdh20 CKO mice after TNBS administration. Weight loss (**a**), colon length (**b**), myeloperoxidase (MPO) activity (**c**). **d** Increased FD4 level detected in the serum of Pcdh20 CKO mice with TNBS-induced colitis. Each group n ≥ 3. * p<0.05, **p<0.01 vs. WT.

Fig S11.


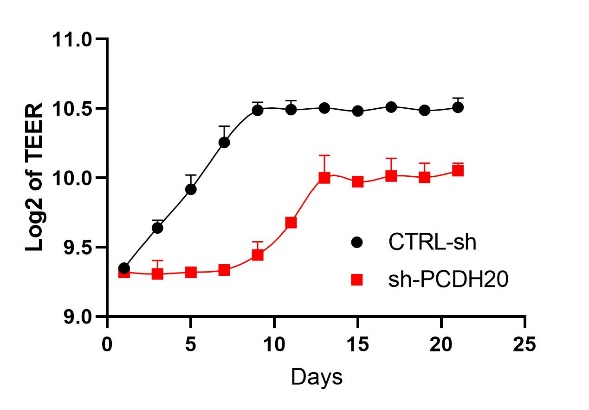


**Fig S11** Log2 of TEER in PCDH20 deficient cell line at different time points.

Fig S12.


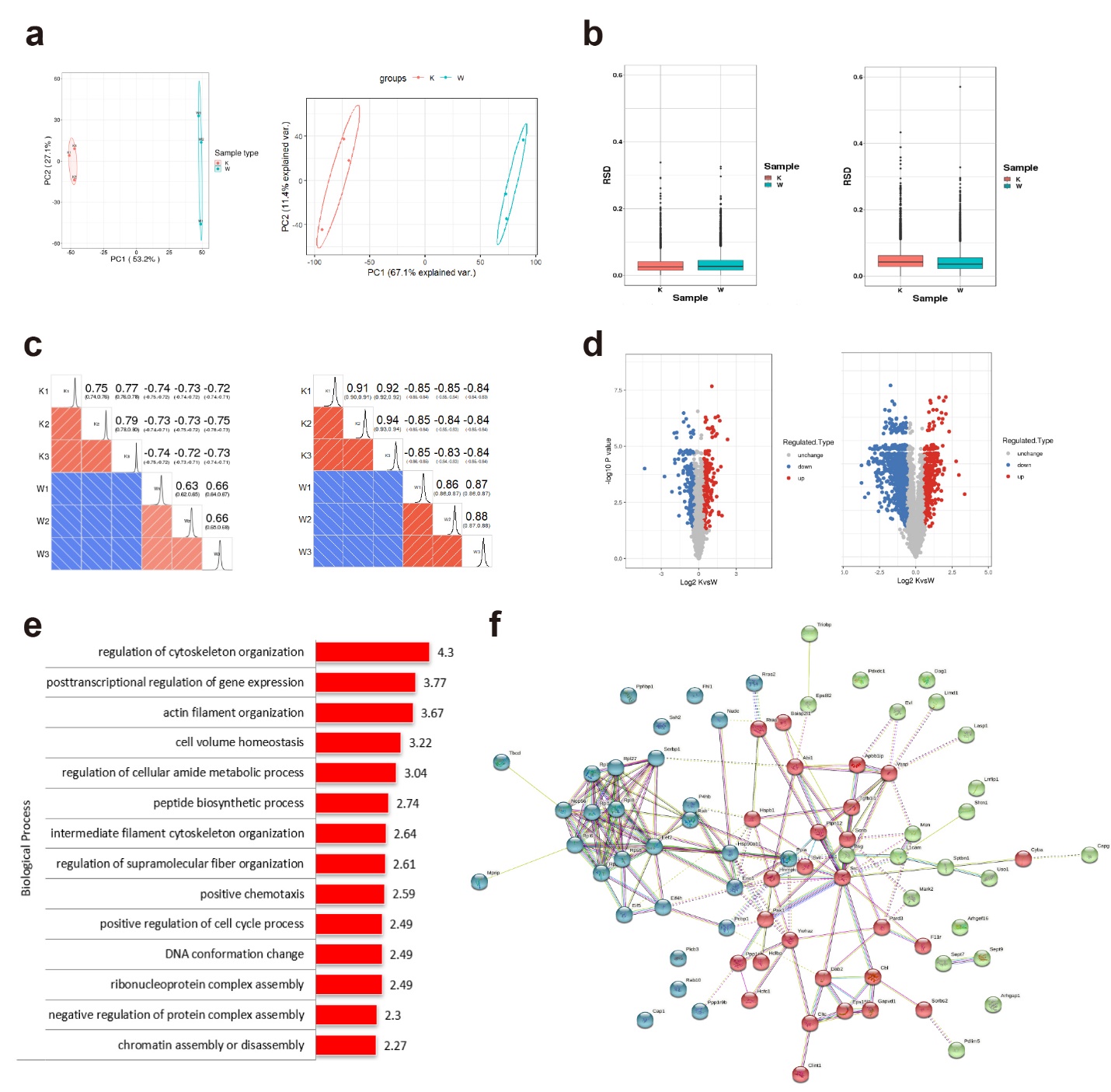


**Fig S12** Proteomics and phosphoproteomics analysis on Pcdh20 CKO mice with DSS colitis. **a-c** Quality control analysis, including principal component analysis (**a**), relative standard deviation (**b**) and Pearson Correlation (**c**). Proteomics (left), phosphoproteomics (right). **d** Volcano plots of proteins with fold change over 1.3 in proteomics (left) and phosphosites with fold change over 1.5 in phosphoproteomics (right). **e** Gene ontology (GO) terms of the top biological process regulated in phosphoproteomics of colon of Pcdh20 CKO mice with DSS-induced colitis. **f** The network of proteins involved in adheren junctions analysed by Cytoscape using data from phosphoproteomics. Each group n = 3.

Fig S13.


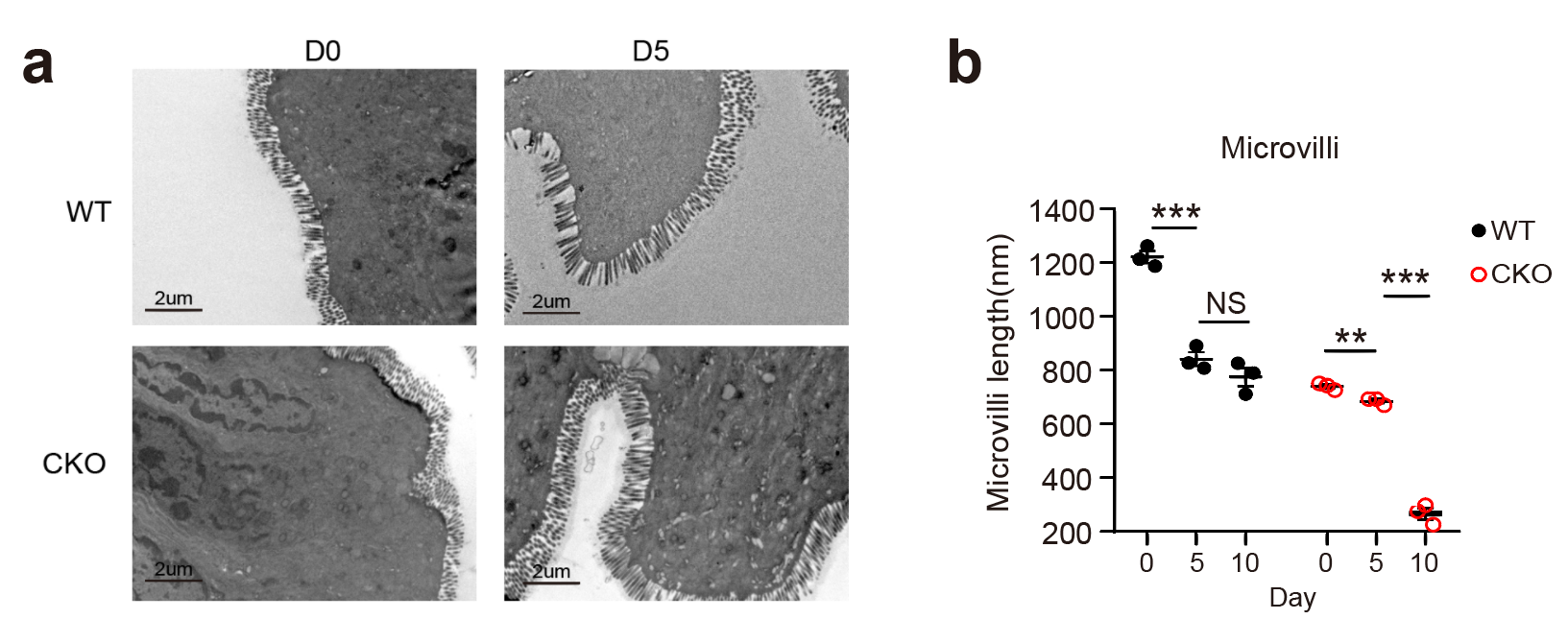


**Fig S13** Adherens junctions in the colon epithelium of Pcdh20 CKO mice with DSS-induced colitis. **a** Adherens junctions observed by transmission electron microscope. Magnification ×3000, scale bars = 2μm. **b** Microvilli length of PCDH20 CKO mice with colitis at different time points.

Fig S14.


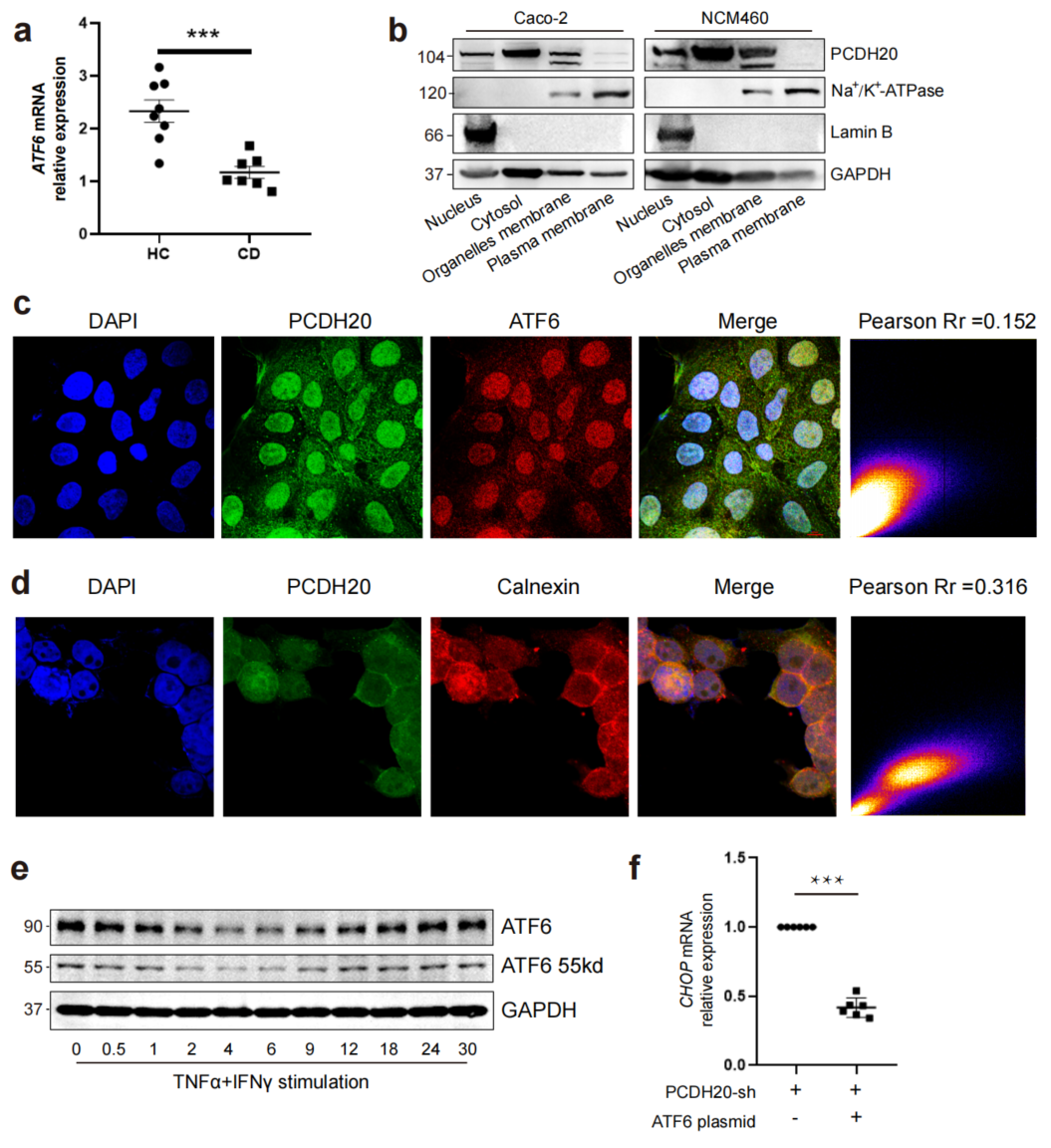


**Fig S14** Cellular location of PCDH20 and expression of ATF6 and its downstream CHOP.

**a** ATF6 expression in the colonic epithelium of PCDH20-deficient CD patients. **b-c** Expression and location of PCDH20 in different cell components, using immunoblot (**b**) and immunofluorescence (**c**). **d** Co-localization analysis of PCDH20 and ER marker Calnexin. **e** ATF6 expression in NCM460 cells with TNFα (2.5 ng/mL) and IFN-γ (10 ng/mL) treatment. **f** Decreased CHOP mRNA level in PCDH20-deficient cell line transfected with ATF6 plasmid. ***p<0.001 vs Control.

Fig S15.


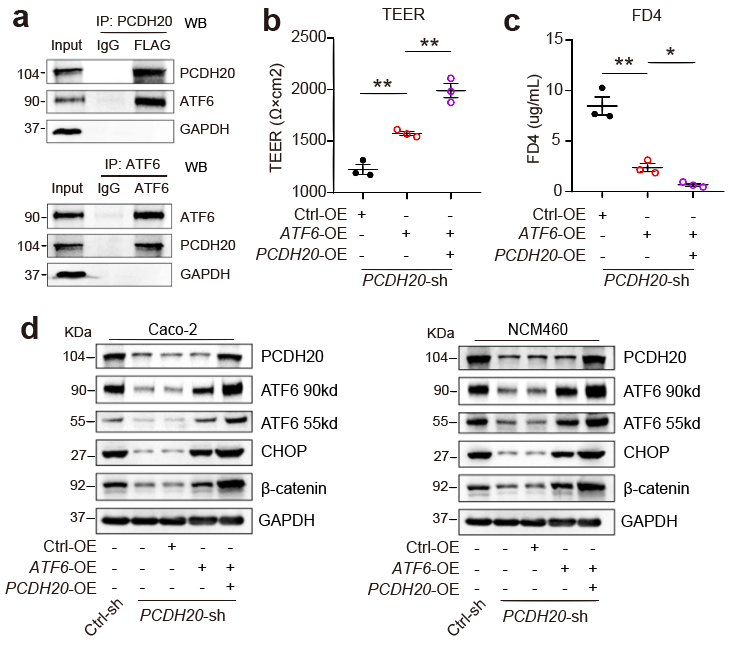


**Fig S15** The interaction and function of PCDH20 and ATF6. **a** Co-immunoprecipitation of PCDH20 and ATF6 in Caco-2 cell line. **b-c** Ameliorated paracellular permeability in PCDH20-deficient Caco-2 cells transfected with different plasmid under inflammatory stimulation (TNFα 2.5 ng/mL, IFN-γ 10 ng/mL). Transepithelial electrical resistance (TEER) **(b**), paracellular permeability assessment with FD4 (**c**). **d** Rescued immunoblots of CHOP/β-catenin in two PCDH20-deficient cell lines transfected with different plasmid under inflammatory stimulation (TNFα 2.5 ng/mL, IFN-γ 10 ng/mL).

Fig S16.


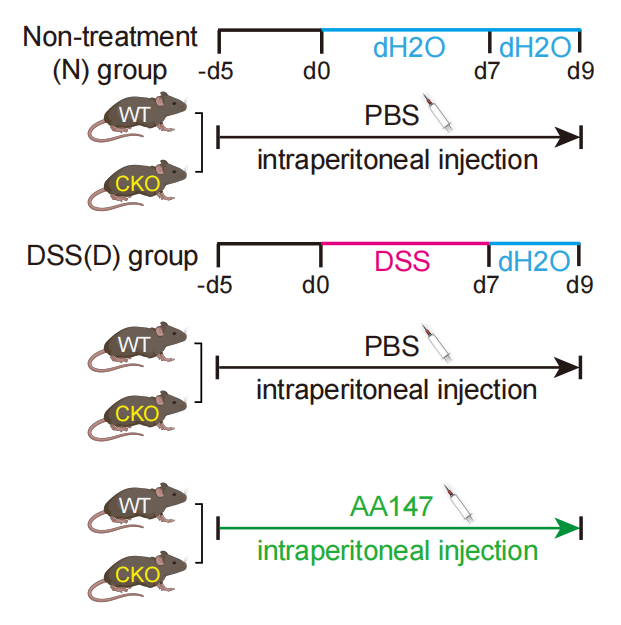


**Fig S16** The experimental design and timeline of AA147 treatment in mice with DSS colitis

Fig S17.


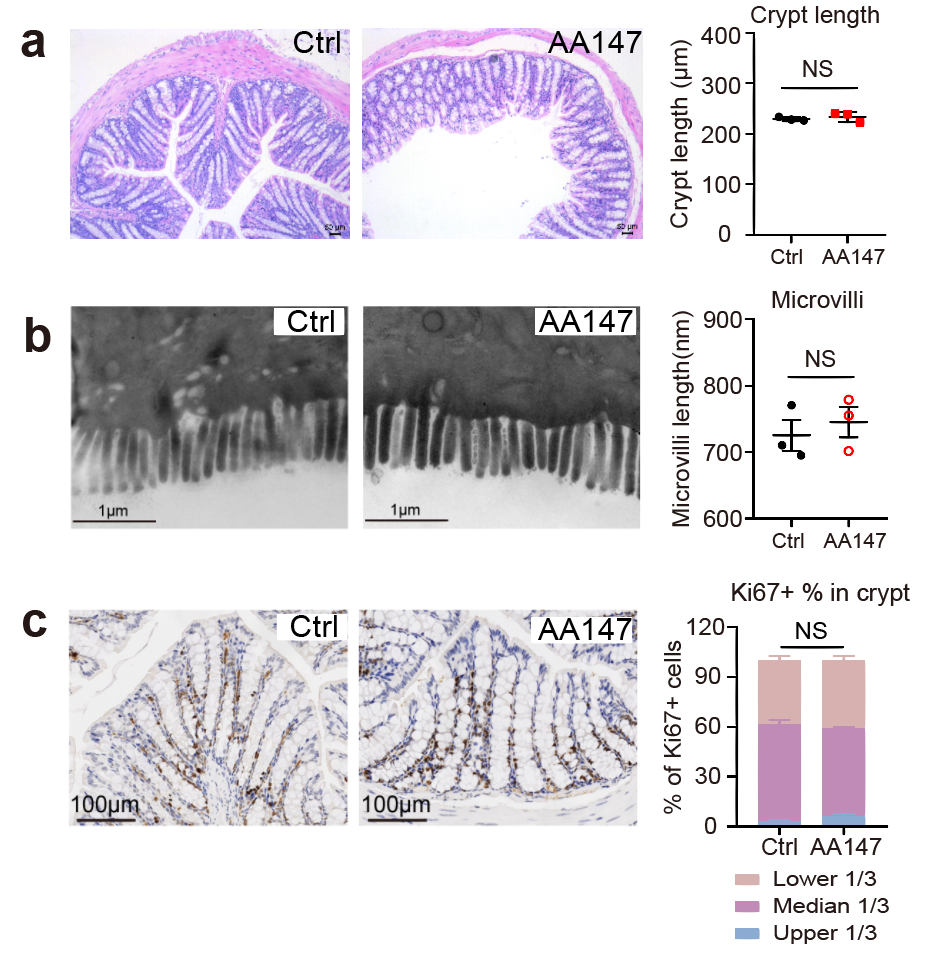


**Fig S17** The effect of AA147 on epithelial morphology and proliferation in Pcdh20 CKO mice. PCDH20 on gut microbiota. **a** Crypt length in colon stained with H&E. Magnification ×100, scale bars = 50μm. **b** microvilli observed with transmission electron microscope. Magnification ×8000, scale bars = 1μm. **c** Representative images of ki67 stained with immunohistochemistry in colon mucosa. Magnification ×200, scale bars = 100μm. Each group n = 3. The data were presented as mean ± SEM. * p<0.05, vs. Ctrl. Intestinal epithelial conditional knockout, CKO.

Fig S18.


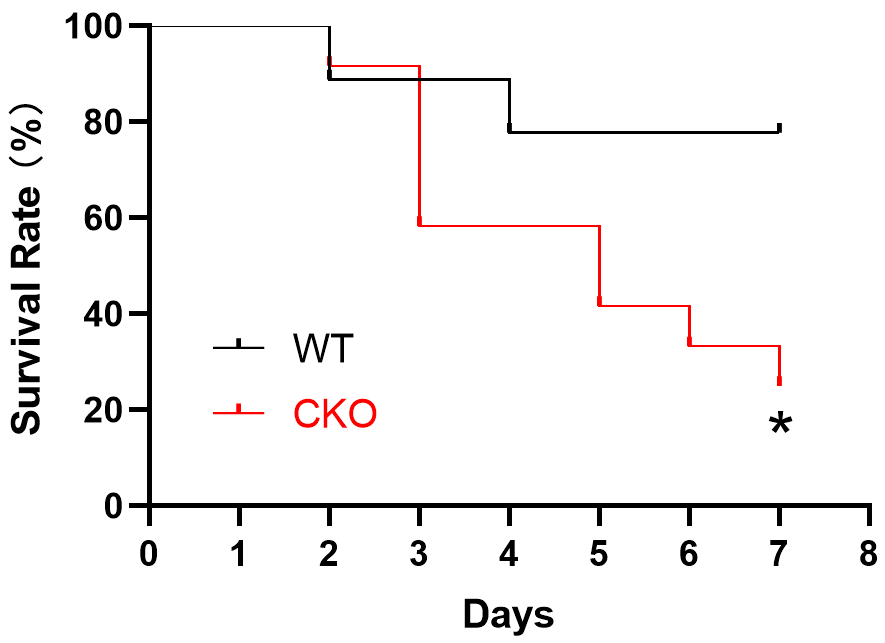


**Fig S18** The survival rate of PCDH20 CKO mice with TNBS colitis

WT n = 9, CKO n = 12, * p<0.05.

Table S1:

The clinical characteristics of Crohn’s disease patients and controls selected for RNA-seq (GSE230113)

|  | CD[n=10] | NC[n=10] |
| --- | --- | --- |
| Gender: Male, *n* (%) | 6 (60.0) | 6 (60.0) |
| Age at diagnosis, *n* (%) |  |  |
| A1 | 0 (0) | / |
| A2 | 7 (70.0) | / |
| A3 | 3 (30.0) | / |
| Location, *n* (%) |  |  |
| L1: Ileal | 0 (0) | / |
| L2: Conlonic | 2 (20.0) | / |
| L3: Ilealcolonic | 8 (80.0) | / |
| Upper tract involvement, *n* (%) | 2 (20.0) | / |
| Disease behavior, *n* (%) |  |  |
| B1: non-stricturing,non-penetrating | 6 (60.0) | / |
| B2: stricturing | 2 (20.0) | / |
| B3: penetrating | 2 (20.0) | / |
| Perianal disease, *n* (%) | 4 (40.0) | / |
| Biospy location, *n* (%) |  |  |
| Transverse colon | 8 (80.0) | 8 (80.0) |
| Descending colon | 1 (10.0) | 1 (10.0) |
| Sigmoid colon | 1 (10.0) | 1 (10.0) |

CD, Crohn’s disease

Table S2:

Clinical characteristics of patients with CD and healthy controls.

|  | HC | CD |
| --- | --- | --- |
| Number of patients | 42 | 43 |
| Gender: |  |  |
| Male | 21 | 28 |
| Female | 21 | 15 |
| Age(y) | 22.95±4.34 | 31.11±9.82 |
| Location |  |  |
| L1: ileal |  | 11 |
| L2: conlonic |  | 6 |
| L3: ilealcolonic |  | 26 |
| Upper tract involvement |  | 9 |
| Disease behavior |  |  |
| B1: non-stricturing,non-penetrating |  | 19 |
| B2: stricturing |  | 15 |
| B3: penetrating |  | 9 |
| Disease duration |  |  |
| Current therapy |  |  |
| 5-aminosalicylates |  | 43 |
| Immunosuppressants/biologics |  | 0 |
