# Supplementary material for: Protocadherin 20 maintains intestinal barrier function to protect against Crohn’s disease by targeting ATF6
Source: Genome Biol. 2023 Jul 5;24:159. doi: 10.1186/s13059-023-02991-0 (PMC10320870; doi:10.1186/s13059-023-02991-0)
Supplement: Supplementary file 4 — Additional file 4. Supplementary methods. [file 13059_2023_2991_MOESM4_ESM.docx]

Materials and Methods

**Evaluation of DSS-induced and TNBS-induced colitis**

Mice were weighed and tested stool consistency along with hemoccult daily to calculate disease activity index (DAI). After mice were sacrificed, colon tissues were fixed and embedded in paraffin for further hematoxylin and eosin staining. The number of neutrophils was semi-quantified by myeloperoxidase (MPO) activity, which was detected by MPO Assay Kit (Jiancheng BioEngineering) following the manufacturer’s protocols. MPO activity was measured in absorbance at 460 nm (SpectraMax M5, Molecular Devices, USA).

**Histopathological analysis**

Histopathological analysis was performed on H&E-stained sections of intestinal samples of Pcdh20 CKO mice aged 8 weeks, as well as these mice at day 10 of DSS colitis. Length of villi and crypts as well as inflammatory bowel disease infiltration, including neutrophil and lymphocytes, were determined on H&E-stained ileal and colonic sections. Pathological scoring of inflammation was evaluated by the depth of tissue damage and inflammatory cell infiltration in lamina propria in a strictly double-blinded manner. Semi-quantified scoring of immunochemistry was evaluated by the shade of color and the stained area in random five views of tissue in a strictly double-blinded manner. Microvilli, adherens junctions and tight junctions were further examined by transmission electron microscope (TEM) (HT7700, HITACHI, Japan), examining for ultrastructure changes.

**Primary Antibodies**

For Western Blotting: primary antibody against PCDH20 (1:1000, LS-C497136, LifeSpan), ATF6 (1:1000, PRS3681, Sigma-Aldrich), α-catenin(1:1000, #3236, CST), β-catenin (1:1000, 610153, BD Biosciences), CDH1 (1:1000, #3195, CST), p120-catenin (1:1000, ab92514, Abcam), phospho T310 of p120-catenin (1:500, ab81318, Abcam), phospho S288 of p120-catenin (1:100, sc-293006, Santa Cruz), CHOP(1:1000, #2895, CST), PERK(1:1000,#5683, CST), GAPDH(1:1000,#5174, CST).

For Immunofluorescence: primary antibody against PCDH20 (1:50, rabbit, PA5-35063, Thermo Fisher Scientific), ATF6 (1:100, mice, ab122897, Abcam).

For Immunohistochemistry: primary antibody against PCDH20 (1:100, sc-84558, Santa Cruz), Ki67 (1:200, ab16667, Abcam), FABP1 (1:200, #13368, CST), Chromo-granin A (1:100, ab254322, Abcam), and DCAMKL1 (1:100, ab109029, Abcam).

**Small hairpin RNA and plasmid transfection**

Lentivirus particles with PCDH20 shRNA or PCDH20 plasmid were purchased from Genechem (Shanghai, China). Cells were transfected with lentivirus particles at 70% confluency. Cells were seeded 24 h prior to transfection with lentiviral particles at an appropriate MOI (MOI 100 for Caco-2 cells, MOI 30 for NCM460 cells) in complete medium and 10 μg/mL polybrene (Invitrogen). Lentivirus containing medium was removed 8 hours later and replaced with complete medium for another 64 hours. Transfected GFP was determined by fluorescence microscope at 72 hours after transfection. Transduced cells were then selected for at least 14 days in a medium with puromycin (100μg/ml for caco-2 cells, 5μg/ml for NCM460 cells) (Invitrogen). On day 17 after transfection, total RNA and protein were extracted to verify knockdown or overexpression of PCDH20.

**Cell Counting Kit-8 proliferation assay**

Cell Counting Kit-8 (Dojindo) was used to quantify the proliferation capability of Caco-2 cell lines with stable PCDH20 overexpression or knockdown. Cells were counted and seeded as 6 replicates with 2,000 cells per well in a 96-well plate. 100ul complete medium containing 10% cell counting reagent was added at 24, 48, 72 hours, respectively. After 4 hours of incubation in a CO2 incubator, the medium was measured for the absorbance on a microplate reader (SpectraMax M5, Molecular Devices) using a 450 nm filter.

**Real-time qPCR**

RNA was extracted using Trizol (Invitrogen) and cDNA was generated using the Transcriptor First Strand cDNA Synthesis kit (Roche). Quantitative PCR was performed using FastStart Universal SYBR Green Master (Roche). Samples were run in a 20 μl reaction mixture as the following PCR program: initial denaturation at 95 °C for 10 min, then 40 cycles of 95 °C for 10 s and 60 °C for 60 s.

Primers:

| **Gene** | **Primer (5'--3')** |
| --- | --- |
| *PCDH20*-human | F: AAGGGTATGCTGAGGGCTAAA |
|  | R: GGAAACAAAACAAGAGGAGGGT |
| *ATF6*-human | F: CTGATGGCTGTTCAATACACAG |
|  | R: GATCCCTTCGAAATGACACAAC |
| *CHOP*-human | F: CTGCTTCTCTGGCTTGGCTGAC |
|  | R: TTGGTCTTCCTCCTCTTCCTCCTG |
| *BETA-ACTIN*-human | F: CTAAGTCATAGTCCGCCTAGAAGCA |
|  | R: TGGCACCCAGCACAATGAA |
| *Pcdh20*-mice | F: GGGTAGCCCTGTCCGTAATG |
|  | R: CCTCCACCCACAGAGTGTAAGA |
| *Beta-actin*-mice | F: GGCTGTATTCCCCTCCATCG |
|  | R: CCAGTTGGTAACAATGCCATGT |

**Protein isolation and Western blotting**

Colon samples were ground by liquid nitrogen into cell power and proteins from tissues or cells were extracted with the appropriate amount of RIPA lysis buffer (Millipore) containing protease inhibitor cocktail (Cell Signaling Technology, CST) in 4°C for at least 20 min. Lysates were centrifuged at 13,000x g at 4°C for 15 min. BCA Protein Assay Kit (Thermo Scientific) was used to estimate the protein concentration of the supernatant. Samples were prepared by using 4× loading buffer (Invitrogen) and boiled for 5 min at 95°C. Gels were prepared by PAGE Gel Fast Preparation Kit (EpiZyme). Samples were separated by electrophoresis in 7.5-12.5% gels and transferred onto the PVDF membrane at 300mA for 90 minutes. Membranes were blocked in TBST containing with 5% (w/v) skim milk powder (or bovine serum albumin for phosphorylated proteins) for 1 hour at room temperature, and incubated overnight at 4°C with 5% BSA in TBST containing primary antibody. The blot was washed three times with TBST and then incubated with HRP-linked secondary antibody (1:2000, CST) for 1 hour at room temperature. The membrane was washed again and incubated with enhanced chemiluminescent HRP substrate (Millipore). All Western blots were performed at least three independent times. Band intensity was quantified analyzed using ImageJ software.

**RNA sequencing and analysis**

For GSE230113, total RNA of colon biopsy from patients with Crohn’s disease and healthy donors was extracted using the TRIzol reagent (Invitrogen, CA, USA) according to the manufacturer’s protocol. RNA integrity was evaluated using the Agilent 2100 Bioanalyzer (Agilent Technologies, Santa Clara, CA, USA). The libraries were constructed using VAHTS Universal V6 RNA-seq Library Prep Kit according to the manufacturer’s instructions. The transcriptome sequencing and analysis were conducted by OE Biotech Co., Ltd. (Shanghai, China). Then these libraries were sequenced on the Illumina Novaseq 6000 sequencing platformand 150 bp paired-end reads were generated. Raw reads of fastq format were firstly processed using fastp software and the low-quality reads were removed to obtain the clean reads. The clean reads were mapped to the reference genome using HISAT2. FPKM of each gene was calculated and the read counts of each gene were obtained by HTSeq-count. PCA analysis were performed using R (v 3.2.0) to evaluate the biological duplication of samples. Differential expression analysis was performed using the DESeq2. *CDH* gene family member with *P* value < 0.05 and foldchange > 1.2 or foldchange < 0.83 was set as the threshold for significantly differential expression gene (DEGs).

For the pulished mRNA microarray sequence dataset (GSE59071), the data was downloaded at [*https://www.ncbi.nlm.nih.gov/geo/query/acc.cgi?acc=GSE59071*](https://www.ncbi.nlm.nih.gov/geo/query/acc.cgi?acc=GSE59071). Differential expression analysis was performed using the DESeq2. Adjusted *P* value < 0.05 and foldchange > 1.1 or foldchange < 0.91 was set as the threshold for significantly DEGs. OriginPro (version 2021) was used to perform hierarchical cluster analysis and heatmap of DEGs.

To find the overlap DEGs of GSE230113 and GSE59071, gene names of DEGs in both datasets were used to perform Venn diagram via OriginPro (version 2021). Hierarchical cluster analysis and heatmap of overlap DEGs in GSE230113 was performed using OriginPro (version 2021).

**Immunofluorescence and immunohistochemistry**

Paraffin-embedded sections were deparaffinization/rehydration by using xylene, gradient ethanol, and dH2O. Antigen unmasking was conducted by sub-boiling slides in citrate solution using a microwave. Cool the slides for 20 minutes.

For immunofluorescence: Slides were incubated in PBST containing 0.1% Triton-X at room temperature for 20 minutes, and then were blocked in PBST containing 5% (w/v) BSA. Slides were incubated overnight at 4°C with PBST containing primary antibody. After three washes with PBST, slides were incubated with secondary antibodies (1:500, AF488 A-21206, AF555 A-31570, AF647 A21241, Invitrogen) in PBST for 1 hour at room temperature. The nucleus was stainied with DAPI (Sigma-Aldrich). Images were captured using the fluorescence microscope (BX63, Olympus) or laser scanning confocal microscope (C2, Nikon). The fluorescence intensity of images was quantified using ImageJ software (Version1.48).

For immunohistochemistry: Incubate sections with 3% hydrogen peroxide for 30 min. The experimental condition of antigen blockage, primary and secondary antibody (see Supplementary Material) incubation were the same as immunofluorescence. Primary antibody. DAB (CST) was applied to slides with appropriate reaction time. Slides were counterstained with hematoxylin and dehydrated before sealing. Images were captured by microscope with bright field (BX63, Olympus). Digital images were obtained using cellSens Dimension (Version 1.8). The quantificaiton of stained cells were counted in random five views of tissue in a strictly double-blinded manner. The IOD value was calculated by using Image-Pro Plus(Version 6.0).

**Fecal Metagenomics**

Briefly, the feces of 6-8 weeks old SPF-grade WT mice and *Pcdh* CKO mice were collected, DNA was extracted, qualified DNA samples were detected, library construction and library detection were performed, and the library that passed the test would be sequenced using Illumina PE150, and the raw data obtained from sequencing would be used for subsequent information analysis.Microbial metagenomic sequencing were respectively performed on the samples, followed by Spearman correlation analysis of differential flora, and the correlation analysis was calculated using the cor function of R software, and the significance test of correlation was calculated using the corPvalueStudent function of the WGCNA package of R software. Data with correlation |r| >= 0.8 and p-value <0.05 were selected. The above bioinformatics analysis was performed by Maiwei Metabolism Company.
