# Supplementary material for: Protocadherin 20 maintains intestinal barrier function to protect against Crohn’s disease by targeting ATF6
Source: Genome Biol. 2023 Jul 5;24:159. doi: 10.1186/s13059-023-02991-0 (PMC10320870; doi:10.1186/s13059-023-02991-0)
Supplement: Supplementary file 5 — Additional file 5. Peer review history. [file 13059_2023_2991_MOESM5_ESM.docx]

**Review history**

**First round of review**

**Reviewer 1**

The authors focus on cadherins given their role in cell-cell adhesion and barrier maintenance, and in particular on PCDH20 which they identify as downregulated in CD, particularly in inflamed tissue. They go on to generate animals with a conditional deletion of PCDH20 in epithelial cells (Vil-Cre), which show defects in epithelial proliferation and potentially differentiation at steady state. These animals also appear more susceptible to DSS and TNBS-induced colitis.
Through a combination of omics and TEM, they suggest that differences in adherens junction and catenin signaling underlie this susceptibility, and further implicate ER stress and ATF6 and CHOP in mechanisms involved downstream of PCDH20. In particular, they show that AA147, an ATF6 activator, rescues the susceptibility of CKO mice to DSS-induced colitis.

The core observation of a novel role for PCDH20 in intestinal biology and during colitis is interesting and will be of interest to the field. However, the work as presented suffers from multiple issues in both reporting, image analysis and mechanistic details that should be addressed before publication.

Major points:
- While the mouse phenotypes presented in the conditional KO and cell lines are of interest, how they relate to each other remains unclear. Multiple lines of evidence suggest that PCDH20 leads to proliferation defects, is this proliferation defect underlying the TEER differences (authors mention doing all TEER experiments at 21d, but if KO lines are proliferating more slowly, the monolayer may not be complete at the same timepoint across conditions)? More importantly, given the phenotype in unperturbed mice, is AA147 acting by modulating DSS severity, or would it also restore the phenotypes described in Fig 2a-d in the absence of DSS treatment?

- The investigation of ATF6 is incomplete as the authors only present data for the 90kD form of the protein, but not the cleaved form that is actually involved in the UPR response and is the one induced by AA147 (e.g. see PMID 29440509). The direct association of PCDH20 and ATF6 is only shown by IP in overexpressing cell lines, or fractionation without fraction specific controls (S9b), and it is unclear whether this association has a true functional role. Figure 6 presents a mechanism where PCDH20's sole role is regulation of ATF6 in the ER, but given the known role of cadherins in cell-cell adhesion, it seems more likely that it would directly regulate junctions

- Finally, the manuscript suffers from major issues in data reporting and analyses. As a few examples, the authors describe performing their own RNAseq in figure 1a, but that data is not mentioned in methods, and only links to GEO/GTEX are provided in the "availability" section. DataS1 only shows expression of a few genes, but not the actual mRNAseq dataset, and the supplemental methods mention using an miRNA (not mRNA) isolation kit ("mirVana miRNA Isolation Kit (Ambion)"). There is no discussion of the statistical approaches used to call differentially expressed genes on either the authors' data or GSE59071. The Pcdh20 mice appear to be newly generated, but are not described beyond saying "generated using the Cre/loxP system" (which exons were targeted, what construct was used to insert loxP sites, requires either a description or a reference). Throughout the text, many images are not analyzed quantitatively (e.g. fig 2d, 2g, 4e would require an additional quantitative analysis across multiple mice).

Minor points:

Fig 1: Fig 1c is mislegended and appears to refer to PCDH20 expression, not pathology score

Fig 2: 2d requires proper quantification across multiple images to support the claim that "Ki67+
proliferating cells in Pcdh20 CKO mice were primarily localized within the median third of the crypt, while those of WT mice were within the lower one third". Similarly, 2g showing differentiation requires quantification, especially for enteroendocrine cell frequency

Fig 3: The methods mention increased mortality of CKO mice in the TNBS model, this should be presented in a figure (how many mice survive past D3, how does this compare to WT)

Fig 4: The related network that should be in figure S7f isn't presented

Statistical approaches are appropriate where described, but they need to be described for RNAseq analysis and image quantification must be added and properly tested where discussed above.

**Reviewer 2**

By integrating RNA-seq data, Huang et al in this manuscript identified PCDH20, a homophilic cell-adhesion codes protocadherin, as a key factor that is downregulated in the colonic epithelium of Crohn's disease (CD) patients and of colitic mice. In conditional knockout mice, loss of epithelial Pcdh20 aggravates intestinal barrier function and colitis severity in vivo and in vitro. PCDH20 deficiency impaired epithelial barrier integrity by unzipping adherens junctions. Finally, they revealed that PCDH20 helped maintain epithelial barrier integrity through the ATF6/CHOP/b-catenin/p-p120-catenin axis. Interestingly, selective activation of ATF6 with AA147 was able to rescue Pcdh20-deficiency-associated epithelial barrier impairment in vivo and in vitro.

Overall, PCDH20 has been suggested to function as a tumor-suppressor gene. Its role in the gut is poorly understood. This manuscript fills that gap by providing the first comprehensive and high-quality exploration of PCDH20 in intestinal epithelial adherens junctions. Most importantly, they have identified PCDH20 as a key factor in gut homeostasis, barrier integrity, and CD development. While the discovery of PCDH20 in CD development is interesting and noteworthy, I do have some additional inquiries that need to be addressed to improve this manuscript.

1. The Method section lacks a detail description regarding the RNA-seq data analysis. For example, how the GSE59071 data were analyzed and how they were integrated with their own RNA-seq data.

2. In addition to the supplemental method, the main Method section also needs a description of RNA-seq as labeled as "mRNAseq by us" in Figure 1a, particularly clinical characteristics of CD patients and controls selected for RNA-seq.

3. The treatment model for Figures 6a-6g should be provided. It should be made clear which group served as the control.

4. The mechanism graph in Figure 6 appears to be fuzzy. Please replace it with a higher resolution version.

5. The labeling of "mRNAseq by us" in Figure 1a is informal. Please revise it.

6. While the study demonstrates the role of PCDH20 in maintaining gut homeostasis, the author may consider discussing its impact on gut microbiota.

7. Some language issues have been observed in the manuscript, it would be beneficial if it could be polished with the professional assistance.

**Authors’ response to reviewers**

Point-to-point reply

Reviewer #1 (Remarks to the Author):
The authors focus on cadherins given their role in cell-cell adhesion and barrier maintenance, and in particular on PCDH20 which they identify as downregulated in CD, particularly in inflamed tissue. They go on to generate animals with a conditional deletion of PCDH20 in epithelial cells (Vil-Cre), which show defects in epithelial proliferation and potentially differentiation at steady state. These animals also appear more susceptible to DSS and TNBS-induced colitis.
Through a combination of omics and TEM, they suggest that differences in adherens junction and catenin signaling underlie this susceptibility, and further implicate ER stress and ATF6 and CHOP in mechanisms involved downstream of PCDH20. In particular, they show that AA147, an ATF6 activator, rescues the susceptibility of CKO mice to DSS-induced colitis.
The core observation of a novel role for PCDH20 in intestinal biology and during colitis is interesting and will be of interest to the field. However, the work as presented suffers from multiple issues in both reporting, image analysis and mechanistic details that should be addressed before publication.
[Response to Reviewer #1]
We truly appreciate the reviewer’s favorable comments and helpful suggestions as well as recognition on the major merits of our work. These suggestions are immensely useful for us to improve the manuscript. We agree with the reviewer on those issues, and we have solved them by performing extra experiments in the revised version.

Major points [1]:
1. While the mouse phenotypes presented in the conditional KO and cell lines are of interest, how they relate to each other remains unclear. Multiple lines of evidence suggest that PCDH20 leads to proliferation defects, is this proliferation defect underlying the TEER differences (authors mention doing all TEER experiments at 21d, but if KO lines are proliferating more slowly, the monolayer may not be complete at the same timepoint across conditions)?
2. More importantly, given the phenotype in unperturbed mice, is AA147 acting by modulating DSS severity, or would it also restore the phenotypes described in Fig 2a-d in the absence of DSS treatment?
[Response 1]
1. We thank the reviewer for the comments. As for the concern on proliferation defect underlaying the TEER differences, all cell lines, including PCDH20 deficient cell line, were complete at the 21st day for TEER examination. Additionally, though different cell lines vary in proliferation rate, the TEER value becomes stable once the monolayer completes (Science. 2018;359:1161-1166). Therefore, the time point of completed monolayer would not affect TEER differences. To further confirmed the effect of monolayer complete timepoint on TEER, PCDH20-deficient and Ctrl-shPCDH20 cell line were seeded, and TEER was tested every two days. As shown in Additional file 1: Fig. S12, monolayer of Ctrl-shPCDH20 cell line completed at day 9, while that of PCDH20 deficient cell line completed at day 13. Yet both cell lines maintained stable TEER value till day 21, suggesting that the results of TEER might not be interfered by proliferation defect in PCDH20 deficient cell line. We have added this finding to the Result section (p.12, line 262-263).
2. We agreed with the reviewer that AA147 rescued Pcdh20 CKO mice with DSS colitis by modulating colitis severity, as showed in Fig 6a-f. AA147 is a small molecule selectively activating ATF6 (Elife. 2016;5:e15550). It has been reported that ATF6 has multiple functions, like regulating ER stress (Inflamm Bowel Dis, 2019, 25(4), 661-671), protecting against endothelial barrier (Cell Signal, 2022, 99, 110432), promoting proliferation (Genes Dis, 2022, 9(4), 981-999; J Allergy Clin Immunol. 2018 Jan;141(1):439-442.e4.), promoting cell differentiation (Sci Signal, 2018, 11(517)) and so on. Nevertheless, activation of ATF6 could reduce cell proliferation and intestinal epithelial stemness in colorectal cancer (Cell Death Dis. 2019;10(7):490). To further figure out the role of AA147 in the defect intestinal phenotype of Pcdh20 CKO mice, we performed extra experiments. Six- to eight-week-old Pcdh20 CKO mice were administered intraperitoneally with AA147 (0.2mg/ml) or for 14 days. As shown in Additional file 1: Fig. S17, in the absence of DSS treatment, no significant differences were observed in colon crypt length, microvilli length, or distribution of Ki67+ cells between the AA147-treating group and the control group. These data suggest that AA147 could not rescue defect of intestinal morphology and distribution of Ki67+ proliferating cells in crypts, which attributed to intestinal epithelial knockout of PCDH20. We also added this finding to the Result section (p. 18, line 381-382). and discussed AA147 in p.23-24, line504-517.

Major points [2]:
1. The investigation of ATF6 is incomplete as the authors only present data for the 90kD form of the protein, but not the cleaved form that is actually involved in the UPR response and is the one induced by AA147 (e.g. see PMID 29440509).
2. The direct association of PCDH20 and ATF6 is only shown by IP in overexpressing cell lines, or fractionation without fraction specific controls (S9b), and it is unclear whether this association has a true functional role.
3. Figure 6 presents a mechanism where PCDH20's sole role is regulation of ATF6 in the ER, but given the known role of cadherins in cell-cell adhesion, it seems more likely that it would directly regulate junctions.
[Response 2]
1. As suggested, we have added new data address the role of ATF6. AA147 is a newly-identified small molecule with capability of selectively activating ATF6 (Elife. 2016;5:e15550). Additionally, AA147 could preferentially increase the expression of ATF6 with a synchronous pattern of both 90kd form and cleaved (55kd) form (Sci Signal, 2018, 11(517); Cell Signal. 2022;99:110432; Front Surg. 2021;8:665260). As the reviewer predicted, we found that the cleaved from of ATF6 expressed consistently with the 90kd form of it, and this pattern became especially obvious with the condition of inflammatory stimulation. As suggested, the 55kd form of ATF6 was added to Fig 5b-c, 5g, 6j, and 6k-l, and these findings were added to the result section (p.15, line 331,333; p.17, line 373-375). We also discussed it in p.23-24, line504-517.
2. We apologized for not providing enough data of direct association between PCDH20 and ATF6, and faction specific controls in Fig S9b (now asS14b). As suggested, extra experiments have been performed. As showed in Additional file 1: Fig. S15a, co-IP confirmed the interaction of PCDH20 and ATF6 in Caco-2 cell line (mentioned in p.16, line 341). Moreover, fractionation with fraction specific controls (Lamin B and Na+/K+-ATPase) was added to Fig S14b, indicating that PCDH20 expressed not only in the plasma membrane but also in the organelle membranes and nucleus. These results suggest that PCDH20 might interact with ATF6 in the ER membrane or the nucleus.
The role of PCDH20 in gut is poorly understood. Recent studies have shown increasing evidence of PCDH family in cell signaling (Science, 2021, 372(6539); Gastroenterology, 2020, 159(6), 2163-2180). We preliminarily found that PCDH20 interacted with ATF6, and proved that PCDH20 maintained epithelial barrier at least partly by interacting with ATF6. To further confirm the functional role of the association between PCDH20 and ATF6, extra experiments were conducted. As shown in the rescue experiments (Additional file 1: Fig. S15b-d), transfection of the ATF6 plasmid partly reversed the decrease in TEER and increase in FD4 in PCDH20-deficient cells compared with those transfected with control plasmid. While PCDH20 overexpression completely rescued the decrease in TEER and increase in FD4 in PCDH20-deficient cells by reviving ATF6 relevant signaling pathway (seen in Additional file 1: Fig. S15b-d). These findings were added to the result section (p.18, line 386-392; p19, line 412-417).
3. Based on our results in Pcdh20 CKO mice, adherens junctions or tight junctions were both intact without DSS treatment (Fig 4d, Additional file 1: Fig. S3d), while a significant gap of adherens junctions was observed in epithelial cells after DSS treatment for 10 days (Fig. 4c, d). Knocking out genes which directly regulate adherens junctions, like Ndrg2, Muc2, and Ctnnd1, canse loosened epithelial adherens junctions without chemical treatment (EBioMedicine, 2020, 61, 103068; Sci Rep, 2020, 10(1), 21135; J Clin Invest. 2010;120(6):1824-35.). The gap of adherens juntions could even be observed as early as birth (J Clin Invest. 2010;120(6):1824-35.). Nevertheless, the gap of adherens junctions only appeared at the late stage of colitis in Pcdh20 CKO mice. It seems that PCDH20 regulated aderens junction by ATF6 mediated signaling pathway rather than directly regulating junctions. We have discussed this issue in the revised text (p.20-21, line.445-452).

Major points [3]:
1. Finally, the manuscript suffers from major issues in data reporting and analyses. As a few examples, the authors describe performing their own RNAseq in figure 1a, but that data is not mentioned in methods, and only links to GEO/GTEX are provided in the "availability" section. DataS1 only shows expression of a few genes, but not the actual mRNAseq dataset, and the supplemental methods mention using an miRNA (not mRNA) isolation kit ("mirVana miRNA Isolation Kit (Ambion)"). There is no discussion of the statistical approaches used to call differentially expressed genes on either the authors' data or GSE59071.
2. The Pcdh20 mice appear to be newly generated, but are not described beyond saying "generated using the Cre/loxP system" (which exons were targeted, what construct was used to insert loxP sites, requires either a description or a reference).
3. Throughout the text, many images are not analyzed quantitatively (e.g. fig 2d, 2g, 4e would require an additional quantitative analysis across multiple mice).
[Response 3]
1. We thank the reviewer for the helpful comments. As suggested, we uploaded the data of our RNAseq to the GEO database and now presented as GSE230113 (https://www.ncbi.nlm.nih.gov/geo/query/acc.cgi?acc=GSE230113). As Data S1 just shows expression of genes included in the heatmap of Fig 1a, we deleted Data S1 in case of misunderstanding. The method and statistical analysis of GSE230113 was added (Additional file 4: Supplementary methods, p.4 para.2-p.5 para 1). We apologized for the misuse of the miRNA isolation kit, and the mistake was corrected in the supplementary method mentioned above. The statistical analysis of GSE59071 was added to Supplementary methods (Additional file 4: Supplementary methods, p. 5, para.2).
2. We agree with the reviewer that Pcdh20 conditional knockout (CKO) mice is newly generated and has not been reported before. To generate Pcdh20fl/fl mice, the exon 3 of Pcdh20-001 gene was targeted and CRISPR/Cas9 system was conducted to mediated loxP sites insertion. As suggested, the detailed description of the Pcdh20fl/fl mice generation was added in the "Method" section (p.25-26, line 553-559).
3. As suggested, quantitative analysis of Fig 2d was added to Fig 2d, that of Fig 2g was added to Fig S5, and that of Fig 4e was added to Fig S13b.
We sincerely thanked the reviewer, as the suggestions helped improving our manuscript immensely.

Minor points:
1. Fig 1: Fig 1c is mislegended and appears to refer to PCDH20 expression, not pathology score
[Response 4] We apologize for the mistake of legend in Fig 1c. In fact, we would like to refer the semi-quantified score of PCDH20 expression. As suggested, the legend has been revised in Fig 1c, and Supplementary Methods. Thank you for this helpful suggestion.

2. Fig 2: 2d requires proper quantification across multiple images to support the claim that "Ki67+ proliferating cells in Pcdh20 CKO mice were primarily localized within the median third of the crypt, while those of WT mice were within the lower one third". Similarly, 2g showing differentiation requires quantification, especially for enteroendocrine cell frequency
[Response 5] We truly appreciate the reviewer’s insightful comments. As suggested, the distribution of Ki67+ cells in the colon crypt was quantified and added to Fig 2d. Moreover, the quantification of cell differentiation in Fig 2g was performed, and was added as Fig S5. Thank you for this helpful suggestion.

3. Fig 3: The methods mention increased mortality of CKO mice in the TNBS model, this should be presented in a figure (how many mice survive past D3, how does this compare to WT)
[Response 6] We truly appreciate the reviewer’s insightful comments. As suggested, the survival rate of PCDH20 CKO mice with TNBS was added to Fig S18, and presented as following. As the figure showed, PCDH20 CKO mice suffered significant higher mortality rate compared to WT mice in TNBS colitis model. Impressively, the survial rate at day 4 in PCDH20 CKO mice was 58.3%, while that of WT mice was 88.9%. Due to the high mortality rate of PCDH20 CKO mice in TNBS colitis model, we sacrificed mice at day3 to prevent data loss.

4. Fig 4: The related network that should be in figure S7f isn't presented.
[Response 7] We apologize for the absence of the related network in Fig S7f (now presented as Fig S12). As suggested, the related network was added to Fig S12f. Brief description was also added to p.13, line 283-284. Thank you for this helpful suggestion.

5. Statistical approaches are appropriate where described, but they need to be described for RNAseq analysis and image quantification must be added and properly tested where discussed above.
[Response 8] In the revised version, RNAseq analysis and image quantification have been added to the method or figures mentioned above.

Reviewer #2: GBIO-D-22-00385 comments:
By integrating RNA-seq data, Huang et al in this manuscript identified PCDH20, a homophilic cell-adhesion codes protocadherin, as a key factor that is downregulated in the colonic epithelium of Crohn's disease (CD) patients and of colitic mice. In conditional knockout mice, loss of epithelial Pcdh20 aggravates intestinal barrier function and colitis severity in vivo and in vitro. PCDH20 deficiency impaired epithelial barrier integrity by unzipping adherens junctions. Finally, they revealed that PCDH20 helped maintain epithelial barrier integrity through the ATF6/CHOP/b-catenin/p-p120-catenin axis. Interestingly, selective activation of ATF6 with AA147 was able to rescue Pcdh20-deficiency-associated epithelial barrier impairment in vivo and in vitro.
Overall, PCDH20 has been suggested to function as a tumor-suppressor gene. Its role in the gut is poorly understood. This manuscript fills that gap by providing the first comprehensive and high-quality exploration of PCDH20 in intestinal epithelial adherens junctions. Most importantly, they have identified PCDH20 as a key factor in gut homeostasis, barrier integrity, and CD development. While the discovery of PCDH20 in CD development is interesting and noteworthy, I do have some additional inquiries that need to be addressed to improve this manuscript.
[General Response to Reviewer #2]
We truly appreciate the reviewer’s favorable comments and recognition on the major merits of our work. These suggestions are immensely useful for us to improve the manuscript.

1. The Method section lacks a detail description regarding the RNA-seq data analysis. For example, how the GSE59071 data were analyzed and how they were integrated with their own RNA-seq data.
[Response 1] As suggested, detail method, analysis, and integation description of GSE230113 (previous as "mRNAseq by us") and GSE59071 was added to Supplementary methods (Additional file 4: Supplementary methods, p.5 para.1-3). Thank you for this helpful suggestion to improve our manuscript.

2. In addition to the supplemental method, the main Method section also needs a description of RNA-seq as labeled as "mRNAseq by us" in Figure 1a, particularly clinical characteristics of CD patients and controls selected for RNA-seq.
[Response 2] The detail method description of GSE230113 (previous as "mRNAseq by us") was added to Supplementary methods (Additional file 4: Supplementary methods, p.4 para.2-p.5 para 1). More importantly, CD patients and healthy controls was selected by a paired manner, and the clinical characteristics was provided in Supplementary Table S1. Thank you for this kind suggestion to improve our manuscript.

3. The treatment model for Figures 6a-6g should be provided. It should be made clear which group served as the control.
[Response 3] We truly appreciate the reviewer’s constructive comments. As suggested, the treatment model for Fig 6a-6g was provided as Fig S16, and the control group was marked in Figures 6a-6b. Thank you for this helpful suggestion to improve our manuscript.

4. The mechanism graph in Figure 6 appears to be fuzzy. Please replace it with a higher resolution version.
[Response 4] As suggested, the mechanism graph in Fig 6m was replaced with a higher resolution version. Thank you for this helpful suggestion.

5. The labeling of "mRNAseq by us" in Figure 1a is informal. Please revise it.
[Response 5] As suggested, the informal label was corrected as “GSE230113” in Fig 1a.

6. While the study demonstrates the role of PCDH20 in maintaining gut homeostasis, the author may consider discussing its impact on gut microbiota.
[Response 6] To study the impact of PCDH20 on gut microbiota, the feces of 6-8 weeks old SPF-grade WT mice and PCDH20 intestinal epithelium-specific knockout mice (3 each group) were collected for metagenomic sequencing. Method of metagenomic sequencing and analysis were added to the Supplementary method part.
As Additional file 1: Fig. S7a-d shows, metagenomic analysis showed that at the species level, knocking out PCDH20 decreased the relative abundance of Bacteroides sp.CAG:927 and Prevotella sp.CAG:485, while increasing the relative abundance of Akkermansia muciniphila, Pseudomonas, Fidobacteria, and Lachnospira A4. Alpha and beta diversity analysis found that PCDH20 deletion deregulated the gut microbiota (Additional file 1: Fig. S7e-f).
To seek gut microbial species markers in PCDH20 CKO mice, LEfSe analysis was used to further identify fecal microbiota that differed significantly between the two groups. The top ten microbial flora contributing to the intestinal microecological environment of PCDH20 CKO mice were Clostridiales, Firmicutes, Clostridium, Lachnospiraceae, Unclassified Firmicutes (f), Lachnospiraceae bacterium A4, Unclassified Firmicutes (c), Unclassified Firmicutes (o), Alistipes, and Lachnospiraceae bacterium 10_1 (Additional file 1: Fig. S8a). Further, we found that Lachnospira, Ruminococcus, Unclassified Clostridium, Clostridium, and Unclassified Firmicutes contributed the most to the dysbiosis after PCDH20 deletion (Additional file 1: Fig. S8b). Based on the abundance of species with differences between groups screened by LEfSe above, a clustering heatmap was shown at the genus level (Additional file 1: Fig. S8c).
KEGG enrichment pathway analysis showed that intestinal microbial differentially expressed genes in Pcdh20 CKO mice were mainly enriched in several signaling pathways such as biosynthesis of other secondary metabolites, amino acid metabolism, lipid metabolism, gene transcription, and membrane transport (Additional file 1: Fig. S9).
These data show that PCDH20 maintained the balance of gut microbiota. We have added these findings in both the result section (p.10, line211-226) and the disecussion section (p.10, line211-226).

7. Some language issues have been observed in the manuscript, it would be beneficial if it could be polished with the professional assistance.
[Response 7] Thank you for this very helpful suggestion to improve the manuscript. As suggested, the manuscript has been polished with the professional assistance by Editage in the revised version.

**Second round of review**

**Reviewer 1**

The authors have addressed all my experimental and technical comments, congratulations on a comprehensive revision.

The only additional suggestion I would make, which can be addressed by text changes, would be to give some context to the finding that Pcdh20's role in colitis, but not homeostasis, is rescued by ATF6 activation.
Currently, the discussion mentions : "Yet, without inflammation, AA147 did not affect defects of intestinal morphology and Ki67+ cells, which attributed to intestinal epithelial knockout of PCDH20.", which is a bit confusing since the colitis susceptibility also depends on epithelial knockout of PCDH20.

The authors should mention either that PCDH20 might have independent contributions to intestinal biology (an homeostatic role independent of ATF6, and a role in inflammation dependent on ATF6), or that short term ATF6 activation might not be sufficient to rescue defects established during early life, or any other explanations. This does not need to be addressed experimentally here, but it is worth discussing in a few more sentences.

**Reviewer 2**

I have no more questions.
